# Optimisation of Flexible Forming Processes Using Multilayer Perceptron Artificial Neural Networks and Genetic Algorithms: A Generalised Approach for Advanced High-Strength Steels

**DOI:** 10.3390/ma17225459

**Published:** 2024-11-08

**Authors:** Luka Sevšek, Tomaž Pepelnjak

**Affiliations:** Forming Laboratory, Faculty of Mechanical Engineering, University of Ljubljana, Aškerčeva 6, 1000 Ljubljana, Slovenia; luka.sevsek@fs.uni-lj.si

**Keywords:** single-point incremental sheet metal forming, sheet metal bulging, hybrid two-step forming, finite element method, multilayer perceptron artificial neural network, genetic algorithm

## Abstract

Flexibility is crucial in forming processes as it allows the production of different product shapes without changing equipment or tooling. Single-point incremental forming (SPIF) provides this flexibility, but often results in excessive sheet metal thinning. To solve this problem, a pre-forming phase can be introduced to ensure a more uniform thickness distribution. This study represents advances in this field by developing a generalised approach that uses a multilayer perceptron artificial neural network (MLP ANN) to predict thinning results from the input parameters and employs a genetic algorithm (GA) to optimise these parameters. This study specifically addresses advanced high-strength steels (AHSSs) and provides insights into their formability and the optimisation of the forming process. The results demonstrate the effectiveness of the proposed method in minimising sheet metal thinning and represent a significant advance in flexible forming technologies applicable to a wide range of materials and industrial applications.

## 1. Introduction

### 1.1. Incremental Sheet Metal Forming

In sheet metal forming, the initial sheet is formed into the final shape of the product by a specific movement of the tool with a specific shape. In conventional forming processes, the shape of the end product is usually directly determined by the shape of the tool. Such processes include deep drawing and sheet metal bulging [1,2]. In order to increase the degree of flexibility, which allows the forming of different product shapes without changing the equipment used, many new unconventional sheet metal forming processes have been developed. One such group of forming processes are the incremental sheet metal forming (ISF) processes, in which the shape of the workpiece is gradually changed towards the final shape of the product. In incremental sheet metal forming, the deformation is localised under a relatively small tool on a small part of the workpiece [3]. ISF processes can be used as forming processes for prototyping and small series production [4]. The reason for this is the relatively long production cycle but the ability to produce different product shapes with the same tool and equipment [5]. Due to this increased flexibility, incremental sheet metal forming processes are particularly useful in aerospace and automotive industries [6].

ISF processes can be performed with or without support, in the form of a support tool with a specific shape, a support tool with a general shape, or a counter punch. The two main disadvantages of the use of ISF in a wider industrial environment are the reduced shape accuracy and the uneven sheet metal thickness of the finished thin-walled part. The geometric deviation of biomedical implants manufactured with ISF is between 1 and 2 mm, which may require further improvements [7]. In the study by Guglielmi et al. [8], ISF forming Mg-alloy AZ31B to the final shape of a cheek implant was presented, with one of the objectives being a sufficiently uniform distribution of the component thickness. Although Guglielmi et al. [8] have shown that it is possible to achieve uniform thickness in complex biomedical components, the study focused primarily on a single material and did not investigate in detail the influence of different geometric, technological, and material parameters. This limitation represents a significant gap, as the formability and thinning behaviour in ISF can be very sensitive to these parameters, especially to different mechanical properties of different material types. Our research fills this gap by including a wider range of parameters in the predictive models, allowing a more comprehensive understanding of their effects on sheet metal thinning and formability for different materials and geometries. In a study by Lu et al. [9], the forming of a TA1 titanium sheet into a cranial implant, using a fixed pre-produced backing plate, was presented. In the study [9], ISF was performed using a classical tool shape with a hemispherical head and a tool with a rolling ball; it was found that the two types of tools mentioned do not drastically affect the uniformity of the wall thickness of the final part. While Lu et al. [9] provided insights into the effects of tool shapes in the ISF process for titanium sheets, their study was limited by the use of a pre-produced backing plate, which inherently limits the flexibility of their presented forming process.

The simplest form of ISF is the one in which no supporting tool of any kind is used [4]. This simplest form of ISF is called single-point incremental forming (SPIF), where the sheet metal is clamped and the path of a small tool determines the final shape of the product. In contrast to the study by Lu et al. [9], our study focuses on the use of a single, hemispherical tool type, which is standard in SPIF, and avoids the constraints imposed by fixed backing plates. This approach allows us to fully exploit the flexibility of SPIF and to systematically investigate the effects of a broader range of geometric, technological, and material parameters on sheet metal thinning.

Incremental forming is suitable for materials with limited formability, including high-strength steels [3]. Advanced high-strength steels (AHSSs), such as dual-phase steels (DP), are widely used in the automotive industry due to their exceptional formability and weight reduction [10] and the combination of high ductility and strength [11]. The ferrite matrix, known as the ductile phase, and the martensite, which is harder and more brittle, provide strength combined with good ductility [12]. DP steels allow a lower initial wall thickness than mild steels, which for this reason are slowly being replaced in the automotive industry [13].

While most research has focused on aluminium alloys and low-strength steel sheets, our study addresses DP steel sheets, which show significant differences in forming force values compared to other materials [14,15]. Since higher force values are to be expected in the SPIF of HSS steel sheets, including DP steel sheets, a suitable selection of the forming machine must be made. The development of a machine that allows sheet metal bulging and SPIF on harder-to-form materials, with a thickness of at least 1 mm, is one of the main aspects of our future work.

To enable flexible production using the SPIF of thin-walled products of different geometries (from low-alloy and low-carbon steels) with excellent mechanical properties, it is necessary to develop predictive models for the specific output parameters of the analysed forming process. These models must consider the effects of the various technological parameters of the process, the geometrical parameters of the equipment used, and the material parameters of the selected steel grades. It is important to emphasise that models taking into account different parameters of all these types (technological, geometrical, and material) have not yet been presented in the literature. The material parameters in the study presented here belong to DP steels, which offer new possibilities in the production of a small series of thin-walled, mechanically superior components, as well as individually manufactured components. This would open up new possibilities in many industries, including the automotive industry and medical sectors.

ISF has two major advantages: the incremental deformation of the material leads to enhanced formability compared to conventional forming processes and the required forming forces are significantly reduced because only a small fraction of the part is deformed [16]. The main disadvantages of ISF processes, including SPIF, is the low productivity and the less uniform sheet thickness of the final product. SPIF can be performed in several steps, with each step allowing the sheet metal to be formed into an intermediate shape of the workpiece. In the study by Filho et al. [17], it was found (both experimentally and through simulations) that multi-step incremental forming can lead to a lower final sheet thickness and a more uniform thickness distribution, as found in SPIF. While this study highlights the benefits of multi-step forming, it focuses primarily on the thickness results without examining the underlying mechanisms that lead to these results or the broader applicability to different materials and geometries, which is addressed in our study. With the aim of reducing sheet metal thinning and production time, SPIF can be combined with other conventional forming processes, additive technologies, and rapid tooling (RT) [18]. However, these combinations often require specialized equipment and complex setups, which can limit their practicality in certain industrial applications. With the aim of achieving a more uniform sheet thickness of the final magnesium product, the study by Palumbo et al. [19] presented a hybrid two-step forming process consisting of the sequential execution of SPIF and superplastic forming. SPIF makes it possible to achieve a suitable preformed magnesium workpiece, with an appropriate sheet thickness distribution, without the use of special tools or equipment [19]. Although this method is promising, the reliance on superplastic forming introduces additional complexities and limitations, particularly with respect to material-specific behaviour. The study by Jagtap et al. [20] showed a significant influence of the bulging depth and the vertical forming step in SPIF on the sheet thickness achieved after both stages of the two-step forming process. However, this study did not fully investigate the optimisation of these parameters or their interaction with other variables, such as tool size or material properties. For the purpose of our study, we extended SPIF to a hybrid two-step forming process, consisting of the sequential execution of bulging with a hemispherical tool and SPIF, with the focus on parameter interaction and possibilities of optimisation. In an additional bulging process, two main additional parameters need to be considered, namely the bulging depth and the diameter of the hemispherical tool, which were also analysed in the study presented here. In the study by Jagtap and Kumar [21], two-step forming, consisting of bulging and SPIF, was performed. From the results of the study [21], the relationship between the tool size during the bulging phase and the target wall angle at the end of the SPIF, with the aim of minimising sheet metal thinning, is evident. Their findings, while valuable, are somewhat limited in scope as they do not consider a comprehensive range of parameters that could further refine the forming process in both SPIF and hybrid two-step forming. Our research builds on this by including additional parameters, including material and geometrical parameters, and evaluating their combined effects on thinning results.

**Figure 1:**
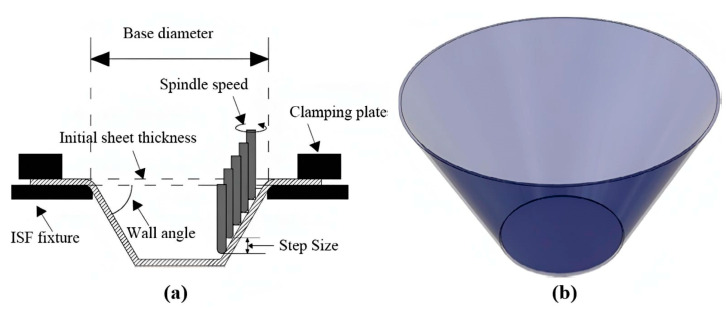
Schematic presentation of (**a**) single-point incremental forming (SPIF) process and (**b**) the shell of a conical part with a fixed wall angle [28].

The fracture that occurs in the workpiece represents the limit for the formability itself and is influenced by the material of the workpiece, as well as technological and geometrical parameters. Figure 1 shows all the important parameters mentioned, as well as the conical shape with a fixed wall angle [22]. This shape, also used in the presented study here, of the final product is ideal for determining the influence of important parameters of the SPIF and the two-step forming, which consists of consecutive execution of bulging and SPIF, on the sheet metal thinning. Increasing the vertical forming step reduces the maximum wall angle of the workpiece up to which SPIF can be performed [23]. On the one hand, increasing the wall angle also has an effect on the thinning during SPIF. On the other hand, the tool trajectory is a parameter in SPIF that not only determines the shape of the final product, but also influences the geometric accuracy of the forming, the residual stresses, the surface finish, and the sheet thickness distribution [24]. Spiral tool paths [25] or, as in the case of our study, paths consisting of levels [26], can be used. In the case of the path consisting of levels, the final shape of the conical part is determined according to the parameters of the part height, the wall angle, and the initial diameter of the conical part shape. The final sheet thickness after SPIF can be approximated using the sine law, which combines the parameters of the initial sheet thickness *t*_0_, the final sheet thickness *t*_i_ and the wall angle of the product *α*. The sine law is given in Equation (1) [27].

(1) $$t_{i}=t_{0}\cdot \sin\alpha$$

### 1.2. Simulation, Prediction, and Optimisation Methods

With the main goal of optimising the forming process of SPIF and hybrid two-step forming, which consists of sequential execution of bulging and SPIF, it is necessary to gain insight into the correlation between key input and output parameters within the processes. For this reason, it is essential to set up and run a sufficient number of simulation models based on the finite element method (FEM). The application of the FEM method makes it possible to obtain a solution that verifies the designs before the physical implementation takes place [29]. The models enable the prediction of the most important output parameter of the study presented here, namely the sheet metal thinning after the forming process in question and after the elastic springback of the workpiece. The variation of selected input parameter values within specific simulation models enables the setting of different prediction models and the subsequent optimisation of the processes.

In our study, artificial neural networks (ANNs) are employed to enhance design efficiency and reduce tooling time, specifically in the context of optimising sheet metal forming processes [30]. To create a predictive value for the important output parameters of a process, including a sheet metal forming process, artificial neural networks (ANNs) can be built. ANN models are generally useful when the mathematical models for a process are not sufficient [31]. ANN models require a data set for learning, which is provided by a sufficient number of experiments or simulations that convert input parameter values into corresponding output parameter values. Trained neural networks memorise the acquired knowledge from the data correlations with which they were trained [32]. Increasing the amount of data on which the neural network is trained does not reliably improve accuracy, but can reduce generalisability [32]. Training the ANN to identify a given input and transfer it to a corresponding output is the task of the training function, which must be chosen correctly to enable fast and accurate predictions by the neural network [32].

**Figure 2:**
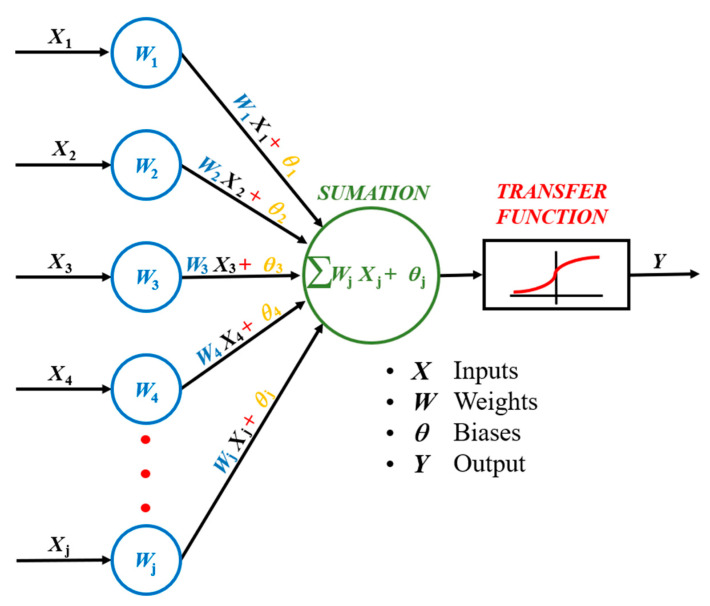
Artificial neural network (ANN) presentation; different *X* parameters represent the inputs, different *W* parameters represent the weights, different *θ* parameters represent the biases, and *Y* presents the final output of the neural network output.

ANNs consist of an input layer, one or more hidden (or concealed) layers, and an output layer [33]. The hidden layers are connected to each other and to the other layers by load, bias, and a transfer function [33]. The most basic computational units in ANN are the neurons [34]. Equation (2) shows the connection between the input layer and the output layer through the hidden layer *h*_i_, where the inputs *x*_j_ pass through the transfer function [35]. The term *θ*_ij_ refers to the bias and *w*_ij_ are the weights that determine the strength of the transfer function [35]. The transfer function calculates the output of each layer by taking the cumulative weights that go into the layer [32]. The type of transfer function used depends on the structure of the neural network itself [32]. Figure 2 shows a schematic representation of an ANN.

(2) $$h_{i}=f\left(\sum w_{ij}\cdot x_{j}+\theta_{ij}\right)$$

One class of ANN is the feed-forward neural network, in which the signal travels only in one direction, from the input layer through the hidden layers to the output layer, while there is no connection between the neurons of the same layer [36]. The back-propagation algorithm is generally used as the learning algorithm [37]. A special type of ANN is the multilayer perceptron artificial neural network (MLP ANN). An MLP ANN is a fully connected feed-forward ANN with at least one hidden layer [38]. Since the MLP ANN is built on the premise of learning the links within the initial data, it is essential to split the data into a training set and a test set [38]. The challenge, however, is to ensure that the model remains generalisable and is not over-fitted to the training data. The prediction accuracy of the MLP ANN set must be evaluated by comparing the predicted values of the output parameters under consideration, with the initial values of the output parameters of the experiments, or simulations created with the same input parameter values. This can be performed by calculating the mean squared error (MSE), the mean absolute percentage error (MAPE), or the coefficient of determination *R*^2^ for each data set used, such as the test set and the validation set, individually or jointly [39].

Zagórski et al. [40] investigated the roughness parameters of the milled surface of a magnesium alloy. The modelling of the relationship between the basic technological parameters of milling and selected roughness parameters was carried out using ANNs, which have proven to be a good tool for predicting surface roughness parameters [40]. However, this study focused primarily on surface roughness and left a gap in understanding how ANNs could be applied to more complex forming processes with multiple, interrelated parameters. In the study by Mekras [31], the focus was on the use of ANN for modelling aluminium-based sheet metal forming processes. An ANN with two hidden layers, each consisting of 12 neurons, was developed for three input parameters and four output parameters [31]. While the ANN model demonstrated its applicability to aluminium in this study, the scope was limited to a few input parameters, and the study did not explore how ANN could be adapted to more sophisticated scenarios with a larger number of variables. In the study by Najm and Paniti [41], the data from 108 components formed with SPIF were collected and ANN was used to determine the material and geometry of the forming tools. The results of the ANN were the formability and geometric accuracy of the components formed with SPIF [41]. Although the study provided valuable insights into tool geometry and material selection, it did not comprehensively address the broader range of factors that influence SPIF results, such as the interaction between geometric, technological, and material parameters. The study by Naga Malleswari et al. [42] focused on a type of 3D printing method, fused deposition modelling (FDM), using ANN and other methods to predict surface roughness, which emphasises the versatility of ANN. In the study by Forcellese et al. [36], ANNs were used to create multi-variable empirical models with the aim of predicting forming limit curves of AZ31 magnesium alloy sheets. The data required for training the neural networks were obtained from tensile testing and biaxial bulge testing [36]. This study has advanced the use of ANNs in the prediction of forming limits, but its focus was limited to a particular material and set of conditions, leaving open questions about how ANNs might work in other forming processes or with other materials. The research by Chan et al. [29] focused on developing an integrated approach to combine FEM and ANN, with the aim of determining the optimal design of the metal-formed product and tooling. A limited number of FEM simulations were used to generate a sufficient amount of data on which the ANN was trained, which was then used for prediction purposes [29]. The integration of FEM and ANN is a promising way to save time and energy. The only concern is the dependence on a limited number of simulations, which affects the generalisability of the model over a wider range of forming scenarios. In the study by Trzepieciński and Lemu [32], the aim was to use an MLP ANN to understand the influence of key friction parameters on the friction coefficient during a drawbead sheet metal forming process. The backpropagation learning algorithm was used for the MLP ANN [32]. In the study by Toros and Ozturk [35], an MLP ANN was used to identify material flow curves of two AL-Mg alloys, namely 5083-H111 and 5754-O, in different temperature ranges. The experimental data from tensile testing were used to train the MLP ANN model [35]. The use of an MLP ANN in the studies by Trzepieciński and Lemu [32] and Toros and Ozturk [35] is limited to certain materials, temperature ranges, and forming processes, so that the adaptability of ANNs of this type to more complex scenarios is questionable. The reason for using an MLP ANN is that multilayer networks have a greater representational capability when dealing with nonlinear, strongly coupled, multivariable systems [35]. Because the presented study is a complex problem of sheet metal thinning during two analysed forming processes, with a large number of interrelated input parameters, the choice of using MLP ANNs is justified. This approach allows us to capture the complicated relationships between the parameters that might be overlooked in simpler models.

When optimising a process, with the aim of minimising or maximising the values of the output parameters of the process itself, many different approaches and methods can be applied. One of these methods is the genetic algorithm (GA), a well-known evolutionary algorithm based on the theory of natural selection and survival of the fittest [43]. A GA uses a kind of roulette to make changes to the values of the input parameters of the process that affect the change in the output parameter under consideration [44]. GA is a commonly used evolutionary computation method that has many advantages in global optimisation problems, such as the lack of premature convergence and reduced computation time [45]. Moreover, a GA does not require gradient computations while it effectively navigates to the global optimum [46].

At each step, the GA randomly selects individual solutions from the current population, assigns them the role of parents, and then uses them to generate ‘children’ or ‘offspring’ for the next population [47]. An individual solution can be evaluated by the fitness function, which can be a simple or complex method with many parameters [48]. A fitness function can also be an appropriately trained ANN, which is then used in a GA [39]. The fitness functions chosen for optimisation with the GA in this study were the properly trained MLP ANNs belonging to the corresponding forming processes analysed and the output parameter of sheet metal thinning. A GA starts by generating a random population of solutions evaluated by the chosen fitness function [49]. A new population is created by selecting parent chromosomes based on fitness, performing crossover, and mutating offspring according to mutation probability, then placing them in the new population [49]. Elite offspring are optimal individuals; crossover offspring are created by combining parent vectors or exchanging information, and mutation offspring are generated by random variations [50]. Each new population is used in the GA [49]. The best solution is provided when the final condition, typically the total number of generations, is reached [49].

Lee et al. [46] focused on the optimisation of grid composite configuration to maximise toughness. While this study successfully optimised material toughness, it was limited to a specific composite configuration, which leaves open questions about the applicability of the approach to more diverse materials and structural challenges, leaving a lot of room for further development in the field of optimisation. In the study by Wei and Yuying [51], a Pareto-based multi-objective GA was used to optimise the deep drawing process for a car body part. The sheet holding force and drawbead restraining force were modified to minimise fractures, wrinkling, insufficient elongation, and thickness variation [51]. The optimisation largely focused on a narrow set of parameters specific to the deep drawing process and it did not provide a more comprehensive approach, optimising a broader range of geometric or even technological parameters. In the study by Liu et al. [50], the problem of elastic springback in sheet metal forming was solved using an ANN and GA. In the study, a GA was used to optimise the weights of the ANN [50]. While the study is an effective way to predict springback, it has its limitations as it focuses on optimising the ANN weights and thus improving the predictive quality of the model without fully investigating the interaction between the different forming parameters. In the study by Tajdeen et al. [49], for electrical discharge machining (EDM), the main input parameters were optimised using a GA to achieve a better material removal rate, lower tool wear rate, and better surface roughness. In the study by Sangwan et al. [39], an integrated feed-forward neural network and GA were used with the aim of determining the optimal machining parameters that produce minimum surface roughness. In the study by Zhao et al. [52], the focus was on ultrasonic welding between a copper wire and aluminium alloy. The analysis was based on an ANN model and optimised by a GA, to investigate the influence of the welding parameters on the joint strength [52]. In the study by Afshari et al. [53], the resistance spot welding of an AZ61 magnesium alloy was analysed and the optimum welding parameters were found using an ANN, integrated with a multi-objective GA. In the study by Savković et al. [54], the possibility of using artificial intelligence methods in milling was investigated. The study analysed the accuracy of three artificial intelligence models built with ANNs, fuzzy logic, and a GA [54]. While the studies by Tajdeen et al. [49], Sangwan et al. [39], Zhao et al. [52], Afshari et al. [53], and Savković et al. [54] have shown the effectiveness of an ANN and GA in optimising various parameters in certain machining and welding processes, they were each limited to a narrow set of parameters and specific applications. While these approaches are successful in their respective fields, they point to a common limitation: the lack of broader applicability across different forming processes. In contrast, our study uses a more comprehensive optimisation framework that integrates AI methods to optimise a wider range of parameters in complex forming processes. This approach not only improves the generalisability of the results, but also provides deeper insights into the interactions between different parameters, making it more relevant and adaptable to a wider range of industrial applications and academic research.

### 1.3. Advances and Scientific Contributions of This Research

Despite significant advances in flexible forming processes such as SPIF and hybrid two-step forming, there are still critical gaps in understanding the collective influence of a comprehensive set of geometric, technological, and material parameters on sheet metal thinning. Previous studies have generally focused on a limited subset of these parameters, meaning that the holistic optimisation of forming processes has not yet been sufficiently explored. The present study addresses these gaps by developing a novel approach that integrates a wide range of parameters into predictive models using MLP ANNs. Our research focuses on accurately predicting the combined effects of these parameters to minimise thinning and optimise forming processes for a variety of materials, especially high-strength steels. We have developed separate MLP ANNs for SPIF and hybrid two-step forming that are specifically designed to predict sheet metal thinning with high accuracy. Furthermore, by incorporating a genetic algorithm, we have created an optimisation framework that allows users to select the desired conical part geometry, material properties, and sheet thickness while optimising the remaining input parameter values with the main goal of minimising sheet metal thinning. This integrated approach represents a significant advance in the field and provides a general solution that improves control over sheet metal thinning and the practical applicability of flexible forming processes.

## 2. Materials and Methods

### 2.1. Parameters of SPIF and Hybrid Two-Step Forming

The analysis in this study focused on the SPIF process and the two-step hybrid forming process, consisting of the sequential execution of bulging and SPIF. The main objective of this study was to determine the change in sheet thickness during the forming of a conical product shape. The question that needed to be answered was which process is better, in terms of minimum sheet metal thinning, when forming a specific conical shape with a specific material of a specific thickness.

**Figure 3:**
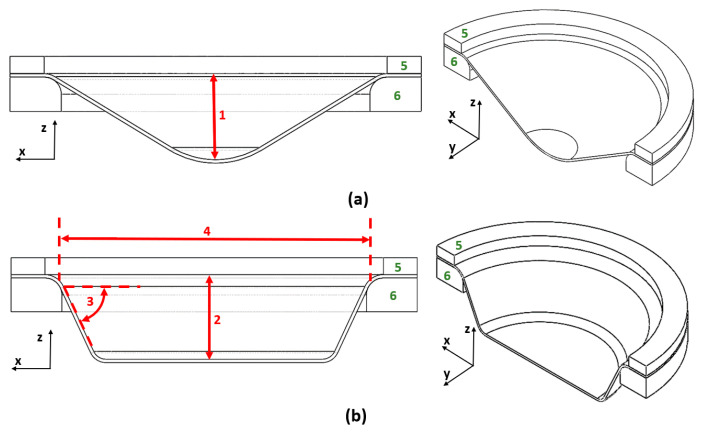
Graphical representation of workpiece shape after (**a**) bulging and (**b**) single-point incremental forming (SPIF). Marked are 1—bulging depth (*H*_bulging_), 2—target workpiece height (*H*), 3—target wall angle (*α*), 4—initial tool path diameter (*D*_path_), 5—blank holder, 6—backing plate.

The most important parameters were selected for the investigated SPIF and hybrid two-step forming with prior bulging and subsequent SPIF. A study by Sevšek et al. [55], in which an influence analysis of the main parameters of the SPIF and additional parameters of the bulging process was carried out, was the basis for this study in terms of selecting the influential parameters, the selection of their suitable values, and the creation of simulation models of the forming processes. Among other things, the study by Sevšek et al. [55] also identified the frequently overlooked parameters that must be included in the analysis of the change in sheet thickness. One of these parameters is the inner diameter of the backing plate, which should be selected accordingly and depends on the initial diameter of the conical workpiece. Three main geometric parameters are required to determine the shape of a conical product. The parameters mentioned are the target workpiece height *H*, the target wall angle of the workpiece *α*, and the initial diameter of the tool path *D*_path_ of the SPIF tool. During the bulging process, it is important to define a suitable value for the bulging depth *H*_bulging_ which, in the case of this study, is carried out with a hemispherical tool. All of the mentioned parameters that determine the tool path of either the bulging tool or SPIF tool are shown in Figure 3. In Figure 3, the two most important parts of the equipment (the backing plate and blank holder) are marked.

The sheet thickness and all of the material parameters used in this study are the essential sheet metal parameters for the analysis of the two forming processes. The material parameters refer to the three essential parameters that determine the flow curve of the material used. The flow curve determines the correlation between the yield stress *σ*_f_, at which the material begins to behave plastically, and the corresponding equivalent plastic strain *ε*_e_. In the case of this study, the aforementioned correlation is given by the parameters of the Hollomon approximation, the strength coefficient *C,* and the strain hardening coefficient *n*, which are given in Equation (3) [56]. Another material parameter tested was the yield strength *R*_p_. The upper and lower limits for the three material parameters tested were determined based on the literature reviewed on different DP steel grades, such as DP780 from the studies by Kumer et al. [10], Han et al. [11], and Schwindt et al. [57], DP500 and DP800 from the study by González-Zapatero et al. [12], DP600 and DP800 from the study by Sodjit et al. [58], and DP980 from the study by Tan et al. [13]. The sheet metal is considered to be isotropic. For this reason, the Lankford parameters of anisotropy in different directions within the sheet surface are considered to be the same. Another parameter that plays an important role in forming is the coefficient of friction *μ* between the SPIF tool, or bulging tool, and the sheet metal.

(3) $$\sigma_{f}=C\cdot {\varepsilon_{e}}^{n}$$

**Figure 4:**
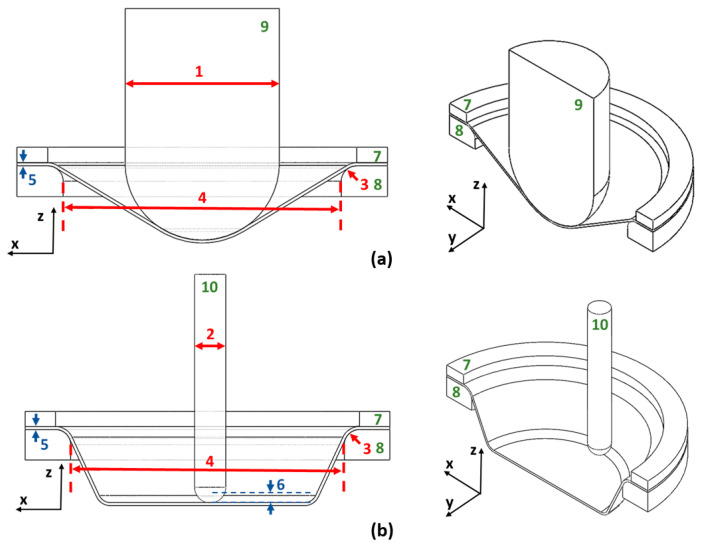
Graphical representation of workpiece shape and equipment used during (**a**) bulging and (**b**) single-point incremental forming (SPIF). Marked are 1—bulging tool diameter (*D*_bulging_), 2—SPIF tool diameter (*D*), 3—backing plate inlet radius (*R*_tool_), 4—backing plate diameter (*D*_tool_), 5—initial sheet thickness (*t*_0_), 6—vertical forming step (*z*), 7—blank holder, 8—backing plate, 9—bulging tool, 10—SPIF tool.

The geometric parameters of the equipment used must not be neglected either. Two main parameters that play a vital role in forming are the geometries of the SPIF tool and bulging tool. Both the tools are hemispherical in shape and are, therefore, defined by the diameter of the bulging tool *D*_bulging_ and the diameter of the SPIF tool *D*, respectively. During SPIF forming, the path of the tool used consists of several planes. The tool is lowered into the next plane by the value of the vertical forming step z, until the desired shape of the part is formed. The backing plate geometry also plays a significant role, considering the geometric parameters of the backing plate diameter *D*_tool_ and inlet radius of the backing plate *R*_tool_. All essential geometric parameters of the equipment used, the initial sheet thickness, and the vital parameter of the vertical forming step are shown in Figure 4.

A large number of simulations, based on the finite element method (FEM), were performed in order to determine a suitable MLP ANN and then optimise the sheet thinning using a genetic algorithm (GA). The main objective of the simulations was to determine the result of sheet metal thinning after forming, followed by elastic springback of the workpiece at the end of either the SPIF or hybrid two-step forming. To create an MLP ANN, numerous simulations were performed with different input parameter values. The input parameter values must be selected from a range of realistic values for a given parameter. Table 1 presents all the parameters used in this study, with their minimum and maximum selected values. The parameter symbol, the parameter name, and the units used are indicated.

For this study, a separate MLP ANN was set for the initial parameter of sheet metal thinning after elastic springback for SPIF and for the same output parameter after hybrid two-step forming. With regard to building the MLP ANNs, 75 simulations of SPIF and 75 simulations of hybrid two-step forming (consisting of a sequential execution of bulging and SPIF), were performed. The 75 simulations of hybrid forming were performed with the same parameter values as the simulations of SPIF, but with two additional parameters that determined the movement and size of the hemispherical bulging tool. These two parameters were the bulging depth *H*_bulging_ and the bulging tool diameter *D*_bulging_. Table 2 shows different combinations of input parameters for the 75 simulations of SPIF and the additional 75 simulations of the hybrid two-step forming process.

### 2.2. Finite Element Method (FEM)

Simulations based on the finite element method (FEM) are used to analyse the forming process. Both the SPIF and the hybrid two-step forming simulations are carried out in the Abaqus simulation environment. The workpiece object is defined as a deformable body and behaves according to the material properties assigned to it. These material properties correspond to the material parameters *C*, *n,* and *R*_p_, which are selected from Table 2 for different simulation models. In this step of building the simulation model, the initial sheet thickness *t*_0_, listed in Table 2 for different simulation models, must be considered. In addition, the dimensions of the equipment, such as the diameter of the SPIF tool *D*, the diameter of the bulging tool *D*_bulging_, the inner diameter of the backing plate *D*_tool_, and the inlet radius of the backing plate *R*_tool_, must be taken from Table 2 for the respective simulation model. An explicit dynamic analysis was performed in the Abaqus simulation environment for all simulation models, as the expected tool movements are high and so the influence of acceleration cannot be neglected. The simulations in the study presented here were carried out using the Abaqus/Explicit simulation environment, with the mass scaling factor kept at 1 and the tool speed adjusted [59].

The contact method used in our study was the kinematic contact method. In order to specify the contact correctly, a certain value of the friction coefficient is determined according to Coulomb’s law of friction. For the study presented here, the coefficient of friction was chosen arbitrarily, within the maximum and minimum values given in Table 1, for a particular simulation model in Table 2.

SPIF forming and hybrid two-step forming, which consists of a sequential execution of bulging and SPIF, are performed with a fixed blank. In the study presented here, a realistic clamping approach was used, in which the 3D object of the blank holder presses on the blank and ensures its fixation [60]. By simulating a more realistic clamping approach, more realistic force values and a more realistic simulation of the forming process can be achieved [61]. During the first step of hybrid forming, the movement of the bulging tool must be set in a straight line, according to the bulging depth value *H*_bulging_, which is given in Table 2 for different simulation models. For SPIF as a stand-alone forming process, or as the second phase of the hybrid forming process, the tool path must be set according to the desired shape of the conical part, which is determined by the parameters of the target workpiece height *H*, the target workpiece wall angle *α*, and the initial tool path diameter *D*_path_. In addition to the parameters *H*, *α*, and *D*_path_, the distance between different levels within the path of the SPIF tool is determined according to the value of the vertical forming step *z*. All parameters that determine the path of the SPIF tool are given for each simulation model in Table 2.

**Figure 5:**
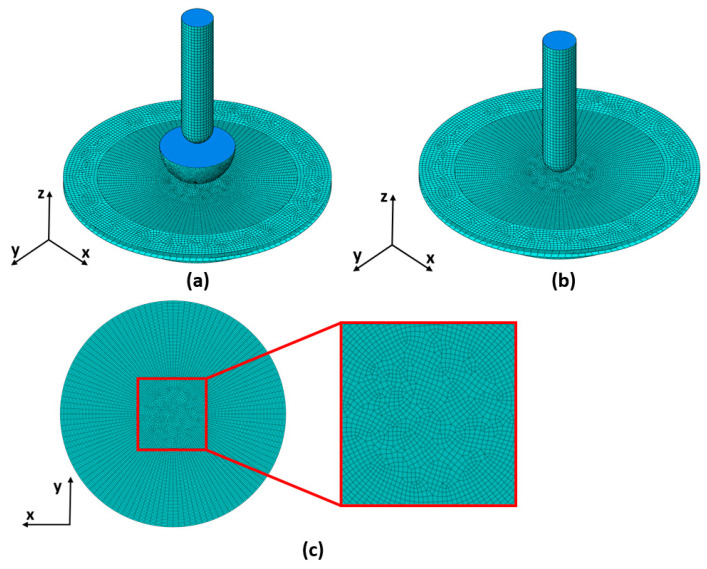
Mesh configurations of finite elements used in the simulation models of the forming processes. (**a**) Meshed assembly for the hybrid two-step forming process, (**b**) meshed assembly for the SPIF process, and (**c**) meshing details of the blank, including a close-up view of the meshed inner section.

With the FEM method, the 3D objects of the equipment and the workpiece must be divided into finite elements. For sheet metal forming processes such as SPIF, shell elements can be used as it is assumed that the sheets are deformed under plane stress conditions [60]. For our study, all rigid 3D objects within the simulation model (i.e., the SPIF tool, the backing plate, the blank holder, and the bulging tool) were meshed with quadrilateral R3D4 elements, while some triangular R3D3 elements were also used for these axisymmetric objects. For the 3D object of the blank or workpiece, square S4R finite elements are used, with some triangular S3 elements used for the inner part of this round object. Figure 5 shows the meshed 3D objects in the hybrid two-step forming assembly and in the SPIF assembly. Since hybrid two-step forming consists of a sequential execution of bulging and SPIF, both the bulging tool and the SPIF tool are positioned in the centre of the blank. Figure 5 also shows the meshing of the 3D object of the blank or workpiece, with a detailed view of the inner section of the object. Table 3 shows the number of triangular and quadrilateral finite elements on different 3D objects of the simulation models used for this study. As the dimensions of certain objects change in the different simulation models from Table 2, the number of finite elements used for these objects also changes. The 3D object of the sheet metal blank is the only one whose dimensions remain the same in all of the simulation models.

### 2.3. Artificial Neural Network (ANN)

The correlation between different geometrical, technological, and material parameters for different simulation models and the corresponding output parameters of sheet metal thinning is the basis for the construction of the artificial neural network. As part of our study, 75 simulations of SPIF and 75 simulations of hybrid two-step forming were carried out. Artificial neural networks were created for the individual forming processes and the individual output parameters. To create an artificial neural network, the data sets must be divided into subsets for training and testing. For our study, a preliminary investigation showed that the data set belonging to the specific output of a specific forming process simulation should be divided into 84% for training and 16% for testing. This means that 63 out of 75 points are used for the training and the rest for the testing. A sufficient number of training iterations, or an epoch of 1000, was performed to ensure that the MLP ANN models converge with the simulations and their conversion from input to output parameters.

Numerous studies have used ANN models with the backpropagation learning algorithm, referred to as the multilayer perceptron (MLP) or multilayer feed-forward algorithm [62]. Equation (4) expresses the multilayer perceptron [41]. The parameter *y* is the output, *x* is the input, *w*_i_ are the weights, and *b* are the biases. The MLP ANN is used for both output parameters in the study presented here.

(4) $$y=f\left(net\right)=f\left(\sum_{i=1}^{n}w_{i}x+b\right)$$

The ANN architecture used in our study is based on the feed-forward multilayer perceptron (MLP) neural network, which is divided into layers. The first layer belongs to the input parameters and the last layer belongs to one or more output parameters. There are one or more hidden layers between these and each layer is made up of neurons. There are as many neurons on the input layer as there are input parameters of the process to be tested. There are 12 input parameters for SPIF and 14 input parameters for hybrid two-step forming in this study. On the output layer, there are as many neurons as there are output parameters for which the neural network was created. In our case, there is one neuron on the output layer that belongs to the specific output parameter of sheet metal thinning, measured in the simulation environment for one of the two tested forming processes. The number of hidden layers and the number of neurons on these hidden layers are the first two essential hyperparameters that determine the architecture of the ANN. For the case of the two tested forming processes in this study, a preliminary investigation was carried out, in which the optimal number of hidden layers was set to five and the optimal number of neurons was determined as a function of the tested output parameters and the corresponding MLP ANNs.

For every mentioned output parameter, an MLP ANN was built with its specific number of neurons on the five hidden layers. In the case of SPIF forming and the thickness change output parameter, the configuration of neurons on the hidden layers was (20,20,20,20,20). In the case of hybrid two-step forming and the thickness change output parameter, the configuration of neurons on the hidden layers was (100,80,60,40,20).

**Figure 6:**
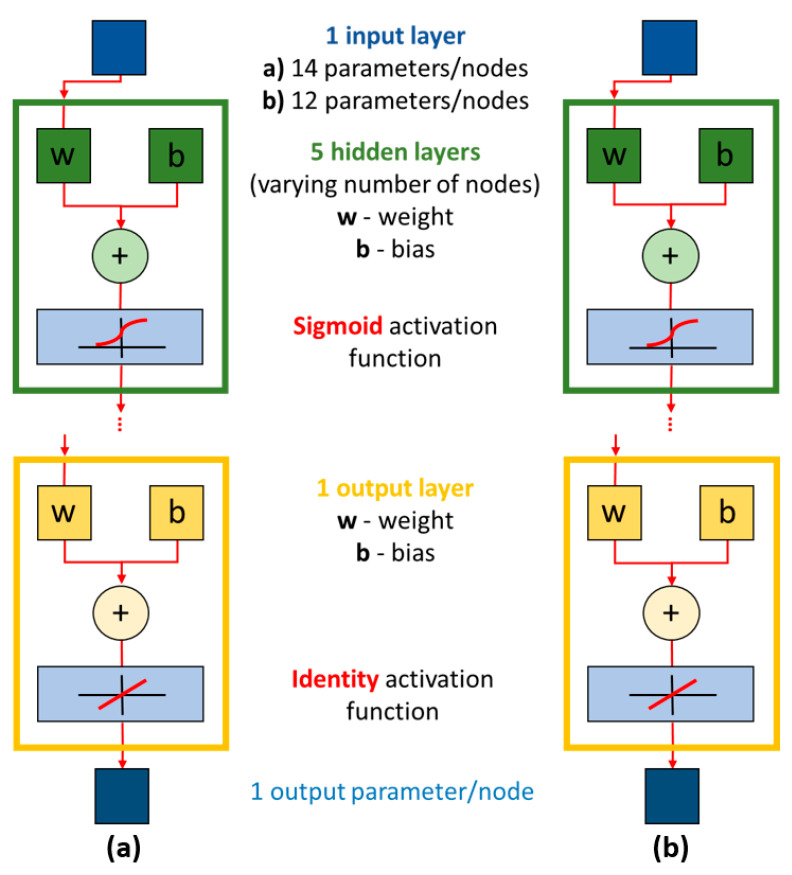
Graphical representation of the Multilayer Perceptron Artificial Neural Network (MLP ANN) models used in this study for (**a**) hybrid two-step forming and (**b**) single-point incremental forming (SPIF).

The ANN structure employed uses standard transfer functions to calculate layer outputs, ensuring accurate predictions for the forming processes analysed. The correct choice of transfer function again depends on the output parameters tested and the corresponding ANNs. The activation function checks the output values, removes certain values (that do not pass the threshold), and maps the remaining values. In the case of the two tested output parameters of both forming processes, a preliminary study found that a sigmoid or logistic activation function can be used for the thickness change output parameter for both investigated forming processes within the hidden layers of their respective neural networks. The logistic activation function produces an output that is proportional to the input. Equation (5) represents the sigmoid or logistic activation function [32]. For the output layer of the two neural networks belonging to the two analysed forming processes, the linear or identity function shown in Equation (6) was used [29]. Figure 6 shows a graphical representation of the two MLP ANNs used in this study and includes all the major layers and a graphical representation of the basic calculation for MLP ANN, which is written in Equation (4).

(5) $$f\left(x\right)=\frac{1}{1+e^{-x}}$$

(6) $$f\left(x\right)=x$$

The MATLAB R2023a software environment was used to define the hyperparameters of the neural network and then train it. For this study, the ‘fitnet’ function was selected in the MATLAB R2023a software, which is suitable for regression tasks and used for problems involving the prediction of a numerical value. The ‘fitnet’ function is also suitable for continuous output variables, i.e., a variable type that can take any numerical value within a certain range, which is suitable for our problem.

It is known that either the Limited-memory Broyden–Fletcher–Goldfarb–Shanno algorithm (LBFGS) or the Adaptive Moment Estimation (ADAM) solver type provides the best possible results for a given initial parameter of the SPIF or the hybrid two-step forming process. The use of the ‘fitnet’ function in the MATLAB R2023a software does not allow the choice of the LBFGS solver, but does allow the choice of the ADAM solver, among others. For this reason, ADAM was chosen for all neural networks in the study presented here. Sufficient prediction accuracy was achieved for all neural networks and these are presented in the following chapters of this paper.

Another hyperparameter within the MLP ANN architecture is the initial learning rate. The value of the learning rate should be chosen carefully, as too small a value slows down the learning process but too high a value may result in an unsteady learning convergence [63]. The initial learning rate values for this study were determined using a preliminary study. The initial learning rate values for the MLP ANNs of the sheet metal thinning output parameters in question were 0.0001 and 0.01 for hybrid forming and SPIF, respectively. The learning rate can be varied during the training process by using the adaptive learning rate, or the learning rate can be constant throughout the learning process [64]. Based on preliminary research, both options were used in our study, namely the adaptive learning rate in the case of SPIF and the constant learning rate in the case of the hybrid forming process.

An appropriate selection of the L2 value for the purpose of two neural networks for the output parameter of sheet thinning, for both SPIF and hybrid two-step forming, was performed in a preliminary study, where different values of the mentioned hyperparameter were tested. The value of L2 was kept low and showed a similar significance in the regression through all the values used as input parameters. The values used for the output parameter of sheet metal thinning were 0.01 and 0.0001, for hybrid two-step forming and SPIF, respectively.

Verifying the correctness of the two MLP ANNs with their hyperparameters is essential before making predictions with them. In the case of the study presented here, the correlation coefficient *R* was calculated directly in MATLAB for all available data together. In addition, the value of the regression coefficient *R*^2^ was calculated for both MLP ANNs, the values of which are shown in the Results section of this paper. The two *R*^2^ values of both sets of MLP ANNs were calculated jointly for the training and testing data. The equation for calculating the *R*^2^ coefficient is given in Equation (7) [38], where $\hat{y}$ represents the predicted output value, *y* represents the actual output value given by the simulation, and *m* represents the length of the vectors $\hat{y}$ and *y*.

(7) $$R^{2}=1-\frac{\sum_{i=1}^{m}{\left(y_{i}-{\hat{y}}_{i}\right)}^{2}}{\sum_{i=1}^{m}{\left(y_{i}-\frac{1}{m}\sum_{i=1}^{m}y_{i}\right)}^{2}}$$

### 2.4. Genetic Algorithm (GA)

One of the main objectives of this study was to minimise sheet metal thinning with the correct choice of input parameter values. For this reason, the genetic algorithm (GA) was chosen. GA works by generating proposed solutions that are packaged in the population, each proposed solution consisting of a list of tested input parameters of our study or chromosomes. The input parameter values are randomly selected from the value ranges defined in Table 1. The evaluation of these randomly selected input parameter values was performed using the fitness function. In our case, the fitness function was an appropriately trained MLP ANN of the corresponding output parameters of the SPIF process or the hybrid two-step forming process.

The basis of the GA is the generation or iteration applied to the initial population. These operations, applied to the existing population, resulted in successive generations of candidates for the input parameter values. These operations are mutation, crossover, and reproduction. Mutation is an operation that causes random changes in different chromosomes [65]. An important parameter of the GA optimisation process is the mutation rate. This parameter refers to the total number of genes within the population and determines the rate at which new genes are introduced into the population and evaluated [65]. In the preliminary study, different mutation rate parameter values were tested and value 0.01 was chosen. Crossover is another operation within the GA. In crossover, two candidate solutions from the existing population are combined and inserted into the next population. The combination of candidate solutions is based on their average chromosome values. The probability that a crossover occurs is the so-called ‘crossover probability’, another parameter that is of great importance in GA. Based on the research results of the preliminary study, the value of the crossover probability was set at 0.9. For this study, the MATLAB program environment was used with default mutation and crossover options, namely ‘mutationgaussian’ and ‘crossoverscattered’. The final operation within the GA is reproduction, which allows the solution to be copied from the current generation to the next.

**Figure 7:**
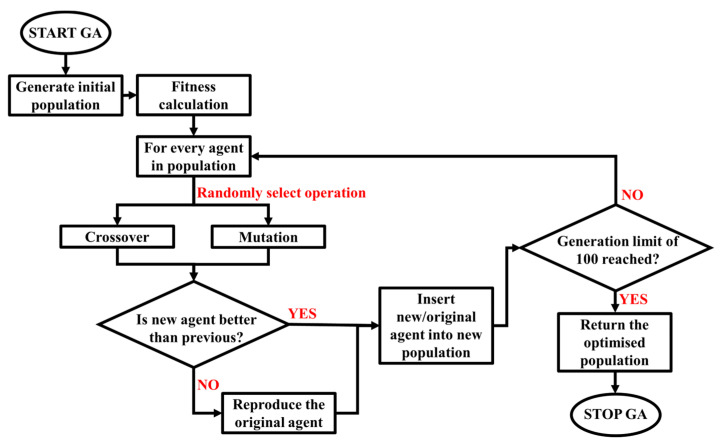
Graphical representation of the genetic algorithm (GA) used in this study.

The main objective of GA was to determine the values of selected input parameters at which the minimum sheet metal thinning occurs during SPIF or the hybrid two-step forming of a desired shape of a conical part from sheet metal with specified material properties. The graphical representation of the GA is shown in Figure 7. The number of generations was set to 100 and the population size to 10.

Therefore, with a properly written programme in MATLAB, based on a GA, the user must be able to enter the values of the parameters that determine the shape of the desired conical product, as well as the parameters that determine the material properties, while all other parameters are optimised to achieve the lowest possible sheet metal thinning. The parameters to be selected by the user should be the target wall angle *α*, the initial tool path diameter *D*_path_, the target workpiece height *H*, the initial sheet thickness *t*_0_, and the material parameter yield strength *R*_p_, strength coefficient *C*, and strain-hardening coefficient *n*. The input parameters to be optimised include the tool diameter *D*, the vertical forming step *z*, the inner diameter of the backing plate *D*_tool_, the entry radius of the backing plate *R*_tool_ and the coefficient of friction *μ*. If the optimisation refers to hybrid two-step forming (which includes preliminary bulging followed by SPIF), then two additional input parameter values must be optimised, namely the bulging tool diameter *D*_bulging_ and bulging depth *H*_bulging_.

The description presented here and the equations of the optimisation process using GA refer to the optimisation within the hybrid two-step forming, in which two additional parameters, namely the diameter of the bulging tool diameter *D*_bulging_ and the bulging depth *H*_bulging_, are optimised compared to SPIF. Additional explanations will show the main differences in optimisation within SPIF. The first step of the optimisation process using GA was to determine the upper and lower bounds of all input parameters corresponding to the values listed in Table 1 for each input parameter. These boundaries were used in the MATLAB environment as the vectors given in Equation (8), for the lower bound or limit ‘*l*’, and in Equation (9), for the upper bound or limit ‘*u*’. As can be seen, the order of the written limits corresponds to the order of the input parameters listed in Table 1. This order of input parameters is used throughout the MATLAB code for the GA. Setting these initial bounds prevents the user from choosing unrealistic values for certain input parameters.

(8) l=[5 1 0.1 30 50 10 50 2.5 20 10 0.05 900 0.14 330]

(9) u=[15 4 1 80 80 40 90 10 50 40 0.20 1400 0.24 760]

In the MATLAB programme environment, a suitable command must be selected to execute the GA algorithm. In MATLAB, the GA algorithm is executed with the command ‘*ga*’ in Equation (10), where *x* corresponds to the input parameters, whose values must be optimised [66]. The values of the input parameters *x* are optimised according to the function ‘*fun*’, which, in our case represents a trained neural network belonging to the specific output of sheet metal thinning, either for SPIF or for hybrid two-step forming. The ‘*fun*’ dimension, or the number of design variables, is written as ‘*nvars*’ and, in the case of this study, is equal to 12 for SPIF forming optimisation and 14 for hybrid two-step forming optimisation. Parameters ‘*lb*’ and ‘*ub*’ refer to the lower and upper bounds within which the input parameters must lie after completion of the GA. Other elements within Equation (10) are discussed below and the values which are specific to this study are presented.

(10) x=ga(fun,nvars,A,b,Aeq,beq,lb,ub)

After entering the user-defined values of the input parameters, constraints must be added to the values of the input parameters, to be optimised with the GA algorithm. For our purposes, the constraints in the MATLAB environment are either linear inequalities or linear equalities, labelled as ‘*A*’, ‘*b*’, ‘*Aeq*’, and ‘*beq*’ in Equation (10). Linear inequalities are written in Equation (11), where ‘*A*’ is a matrix by which the separate input parameter ‘*x*’ is multiplied, and ‘*b*’ is the column vector whose values belong to the results of the inequality. Since there are no inequalities in this study, both ‘*A*’ and ‘*b*’ are set as an empty matrix and an empty vector, respectively.

(11) A·x≤b;A=[ ], b=[ ]

Separately, linear equalities were used, so that the GA algorithm provides the correct values for the optimised and user-selected values of the input parameters. The linear equalities are shown in Equation (12), where ‘*Aeq*’ is a matrix by which the respective input parameter ‘*x*’ is multiplied and ‘*beq*’ is a column vector whose values belong to the equality results. It should be emphasised that, if the parameter needs to be optimised with the GA algorithm, the corresponding values in ‘*Aeq*’ and ‘*beq*’ are equal to 0. If the parameter is selected by the user, the corresponding value in ‘*Aeq*’ is 1 and in ‘*beq*’ the value corresponds to the input value selected by the user.

The target product height *H* is selected by the user, but the value of z must be such that the selected target height of the product *H* is reached exactly. Furthermore, the values of *z* after optimisation can be chosen between 0.1 and 1.0 mm and the values of *H* between 10 and 40 mm, as defined in Table 1. For this reason, a random integer between 40 and 100 is generated in the third row and the third column of ‘*Aeq*’, which makes it possible to optimise the value of *z* and still achieve the forming to the value of *H* chosen by the user. In Equation (12), the vector ‘*beq*’ has 14 elements and the matrix ‘*Aeq*’ has 14 rows and 14 columns, since the optimisation presented here refers to the hybrid two-step forming, with 14 input parameters in this study. In the case of SPIF forming, ‘*beq*’ has 12 elements and ‘*Aeq*’ has 12 rows and 12 columns, as the parameters of bulging tool diameter *D*_bulging_ and bulging depth *H*_bulging_ are not present in SPIF.

(12) $$Aeq\cdot x=beq;\;Aeq=\left[\begin{array}{cccccccccccccc} 0 & 0 & 0 & 0 & 0 & 0 & 0 & 0 & 0 & 0 & 0 & 0 & 0 & 0\\ 0 & 1 & 0 & 0 & 0 & 0 & 0 & 0 & 0 & 0 & 0 & 0 & 0 & 0\\ 0 & 0 & randi([40,100]) & 0 & 0 & 0 & 0 & 0 & 0 & 0 & 0 & 0 & 0 & 0\\ 0 & 0 & 0 & 1 & 0 & 0 & 0 & 0 & 0 & 0 & 0 & 0 & 0 & 0\\ 0 & 0 & 0 & 0 & 1 & 0 & 0 & 0 & 0 & 0 & 0 & 0 & 0 & 0\\ 0 & 0 & 0 & 0 & 0 & 1 & 0 & 0 & 0 & 0 & 0 & 0 & 0 & 0\\ 0 & 0 & 0 & 0 & 0 & 0 & 0 & 0 & 0 & 0 & 0 & 0 & 0 & 0\\ 0 & 0 & 0 & 0 & 0 & 0 & 0 & 0 & 0 & 0 & 0 & 0 & 0 & 0\\ 0 & 0 & 0 & 0 & 0 & 0 & 0 & 0 & 0 & 0 & 0 & 0 & 0 & 0\\ 0 & 0 & 0 & 0 & 0 & 0 & 0 & 0 & 0 & 1 & 0 & 0 & 0 & 0\\ 0 & 0 & 0 & 0 & 0 & 0 & 0 & 0 & 0 & 0 & 0 & 0 & 0 & 0\\ 0 & 0 & 0 & 0 & 0 & 0 & 0 & 0 & 0 & 0 & 0 & 1 & 0 & 0\\ 0 & 0 & 0 & 0 & 0 & 0 & 0 & 0 & 0 & 0 & 0 & 0 & 1 & 0\\ 0 & 0 & 0 & 0 & 0 & 0 & 0 & 0 & 0 & 0 & 0 & 0 & 0 & 1 \end{array}\right],\;beq=\left[0;t;H;\alpha ;D_{path};H;0;0;0;H;0;C,n;R_{p}\right]$$

**Figure 8:**
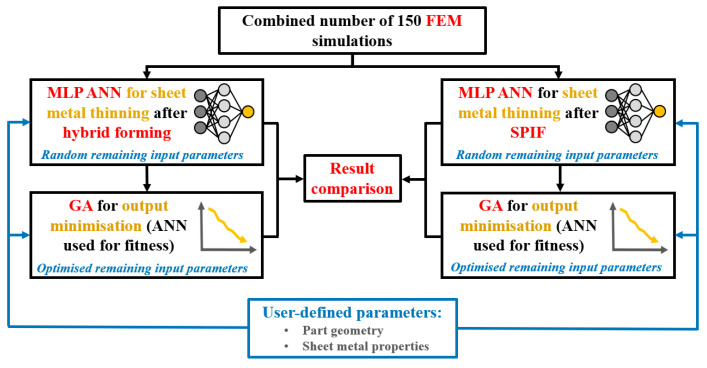
Graphical representation of the research steps followed in this study.

As shown in Figure 7, the objective of the GA is to minimise the output parameter of sheet metal thinning and return the corresponding input parameter values, either for SPIF or for the hybrid two-step forming consisting of the sequential execution of bulging and SPIF. The obtained optimised values of the input parameters can then be fed into the set MLP ANN of the specific forming process and the optimised prediction of sheet metal thinning can be made as the output parameter. In addition, the GA is performed separately for the SPIF process and the hybrid process, with the same input parameter values selected by the user. For a better understanding of the process presented in the study, Figure 8 shows a graphical representation of the research steps in this study. In order to compare the optimised output values of sheet metal thinning for SPIF and hybrid two-step forming, the same non-optimised random input parameters were also fed separately to the MLP ANN of SPIF and the MLP ANN of hybrid two-step forming, as explained in the following chapters.

## 3. Results

### 3.1. FEM Simulations of SPIF and Hybrid Two-Step-Forming

**Figure 9:**
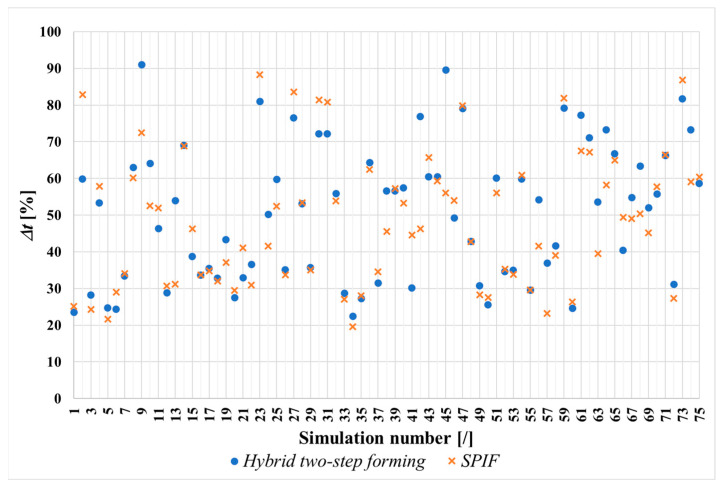
Scatter diagram showing sheet metal thinning (Δ*t*) values for each of the 75 simulations conducted in this study.

The simulation models based on the FEM method were created in accordance with the explanations in Section 2.2. The main objective of the performed simulations of the SPIF and the hybrid two-step forming process was to gain insight into sheet metal thinning. For this purpose, the initial sheet thickness listed for all simulation models in Table 2 was compared with the minimum sheet thickness value achieved at the end of the simulations performed. For the 75 simulations of SPIF and 75 simulations of hybrid two-step forming, with the input parameter values listed in Table 2, the sheet metal thinning results are shown in Table 4. To provide a clearer visualization of the simulation results from Table 4, a scatter diagram illustrating the sheet metal thinning (Δ*t*) for each of the 75 simulations of hybrid two-step forming and SPIF is shown on Figure 9. The x-axis represents the simulation numbers, where the same input parameter values were applied for both hybrid two-step forming and SPIF. This allows for a direct comparison of the results for the two forming processes at each simulation number, highlighting the differences in sheet metal thinning outcomes under identical input parameter values of SPIF as a stand-alone forming process or as the second step of the hybrid two-step forming process.

To verify the simulation results of this study, a convergence analysis was performed, focusing on increasing the mesh density of the 3D blank and tool objects within the simulation models. By increasing the mesh density, the sheet thinning results were verified and their convergence towards a singular value was analysed. Achieving a sufficiently high mesh density, in terms of result accuracy, while not excessively increasing the simulation runtime was the top priority in this study. In this approach, the mesh density and the number of elements was set to the values listed in Table 3. As a more realistic approach for sheet clamping is warranted [60], a similar approach to convergence analysis was performed for the value of blank holder force. A sufficient value of this parameter is required to ensure the fixation of the blank 3D object during all simulations performed but, on the other hand, it should not affect the sheet thinning results obtained. Considering the increase in the speed of SPIF tool movement, which is a common approach in explicit analyses [67], the same convergence analysis was performed for the tool speed during SPIF forming and, thus, for the simulation step time in which SPIF takes place. Stable simulation models for SPIF and hybrid two-step forming were established with all the mentioned convergence analyses. In addition, a sensitivity analysis was performed by varying the main input parameter values and checking the simulation results. This type of analysis was performed with respect to the possible input parameter values listed in Table 1, which are based on the possible values in the reviewed literature. Changing the value of a particular input parameter leads to a change in the sheet thinning as an output parameter of the simulation. The trends in sheet thinning values and thus the effects of the analysed input parameters, were compared with the results of the studies by Mulay et al. [23], Abdelkader et al. [68], Tayebi et al. [25], and Neto et al. [26], which focused on the SPIF process. A preliminary study on the influence of input parameters and their correlation, by Sevšek et al. [55], highlighted the most important input parameters influencing sheet metal thinning, indicating which input parameters the sensitivity analysis should focus on.

An additional validation of the simulation results was performed by comparing the sheet metal thinning results with the results of similar experiments from the literature. For instance, simulations were conducted using the data from Mulay et al. [23] and Bansal et al. [69], which involved SPIF forming of various materials into conical-shaped final parts. These conical shapes are standard for SPIF tests, as they effectively demonstrate the influence of different parameters. In addition, the simulation results were compared with the final sheet thickness values after hybrid two-step forming, consisting of a sequential execution of bulging and SPIF, which is presented in the study by Jagtap and Kumar [21]. The results of our simulations showed minimal discrepancies with these studies, confirming the accuracy of our FEM models. Below, we provide detailed comparisons of our simulation results with the findings from these studies to highlight the robustness and reliability of our models:

1. Mulay et al. [23] validation:
  - Parameters: DC04 steel and AA5754-H22 aluminium, 0.8 mm thickness, 10 mm tool diameter, 0.6 mm vertical forming step, 118 mm initial diameter, 65° wall angle for DC04, 60° for AA5754-H22.
  - Results: The minimum sheet thicknesses were 0.33 mm and 0.35 mm in Mulay et al.’s study, while our simulations resulted in 0.31 mm and 0.35 mm, for both tested materials, respectively.
2. Bansal et al. [69] validation:
  - Parameters: AA5052-0 material, 0.88 mm thickness, target wall angle of 42.5°, tool diameters (8 mm or 12.7 mm), and vertical forming steps (0.1 mm and 0.45 mm).
  - Results: Our simulations showed sheet thicknesses of 0.62 mm, 0.60 mm, 0.79 mm, closely matching Bansal et al.’s values of 0.64 mm, 0.62 mm, 0.63 mm, and 0.80 mm.
3. Jagtap and Kumar [21] validation:
  - Parameters: Al-1050 material, 1.22 mm thickness, 50 mm bulging tool diameter, various wall angles and bulging depths (wall angles of 30°, 50°, and 70°; bulging depths of 10 mm, 14 mm, and 18 mm).
  - Results: The minimum sheet thicknesses from our simulations matched closely with Jagtap and Kumar’s results, showing discrepancies below 5%.

A discrepancy of less than 5% of the achieved sheet metal thinning, between the repeated simulations and the original experimental results from the literature, shows the general correctness of the simulation models for both forming processes in this study.

Since both types of forming processes were performed with the same values of input parameters of SPIF, either as a stand-alone process or as a second step of the hybrid forming process, the two analysed forming processes can be compared first-hand. As can be seen from the simulation results in Table 4, a larger sheet thinning can be present for both the first and the second analysed forming process if the same input parameters of SPIF are used, e.g., the same row in Table 4. A suitable choice should be made between the two forming processes, to reduce sheet thinning when forming any shape of a conical product. In addition, when forming a user-defined shape of a conical product from a specific material, the remaining input parameters should be optimised to achieve minimum sheet metal thinning. The comparison between the forming processes and the optimisation of the input parameter values are discussed in the following chapters of this work.

**Figure 10:**
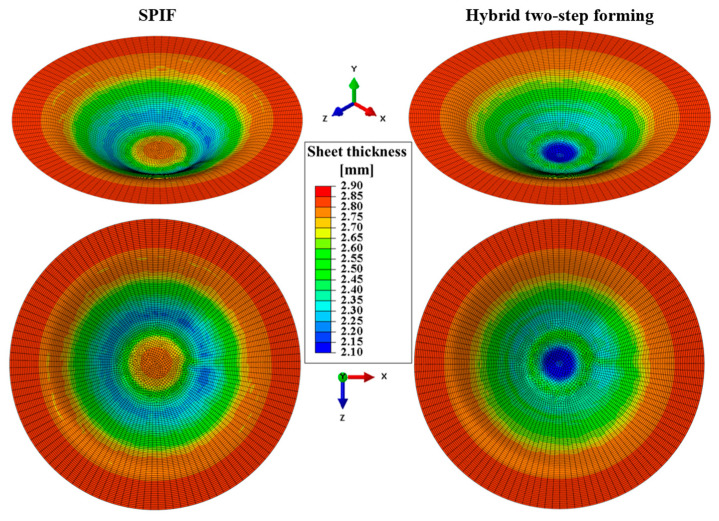
Sheet thickness distribution after the completion of single-point incremental forming (SPIF) and hybrid two-step forming for the fifth set of input parameters from Table 2.

The simulation results clearly show at which section of the product the greatest sheet metal thinning occurs. With SPIF, the greatest thinning occurs in the wall area of the workpiece. When the sheet metal is bulged during the first step of the hybrid two-step forming, the greatest thinning occurs in the middle area of the workpiece, below the hemispherical tool used. This result is in good agreement with the literature [21]. The sheet thickness distribution at the end of the simulations of SPIF and hybrid two-step forming, using the input parameters of the fifth parameter set listed in Table 2, is shown in Figure 10.

As a result of the thinning that occurs at different sections of the workpiece during bulging and SPIF, the hybrid two-stage forming process presented here can be a good choice to minimise sheet metal thinning for certain shapes of conical parts. Further discussion on this topic is provided in the results section of this paper. The FEM simulations performed for this study, using the input data listed in Table 2 and the sheet metal thinning results listed in Table 4, form the basis for creating the MLP ANN models of the two forming processes under consideration. The MLP ANN approach is presented in Section 3.2.

### 3.2. Set-Up and Evaluation of MLP ANN

Using the MLP ANN, a fully interconnected, feedforward ANN whose architecture is determined by properly chosen hyperparameters, we were able to predict the value of a given output parameter for any combination of input parameters. For this study, we employed two artificial neural networks belonging to two output parameters of sheet metal thinning during the forming process of SPIF and hybrid two-step forming. The selected hyperparameter values are listed in Table 5.

**Figure 11:**
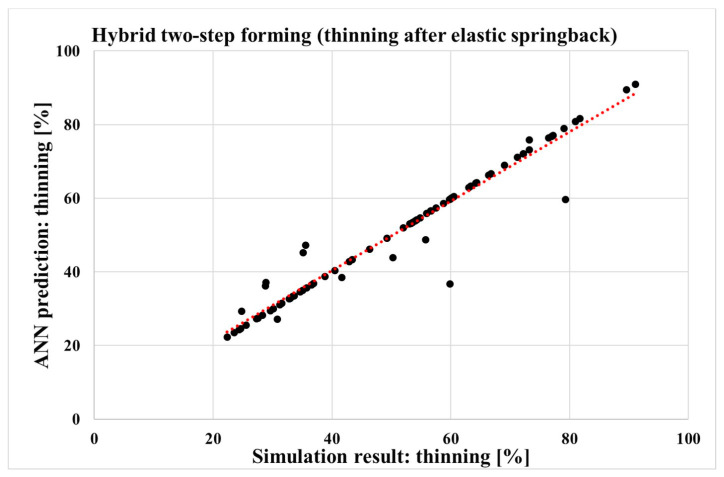
Comparison between the sheet metal thinning after elastic springback as predicted by the Multilayer Perceptron Artificial Neural Network (MLP ANN) and the thinning values obtained from simulations for hybrid two-step forming.

**Figure 12:**
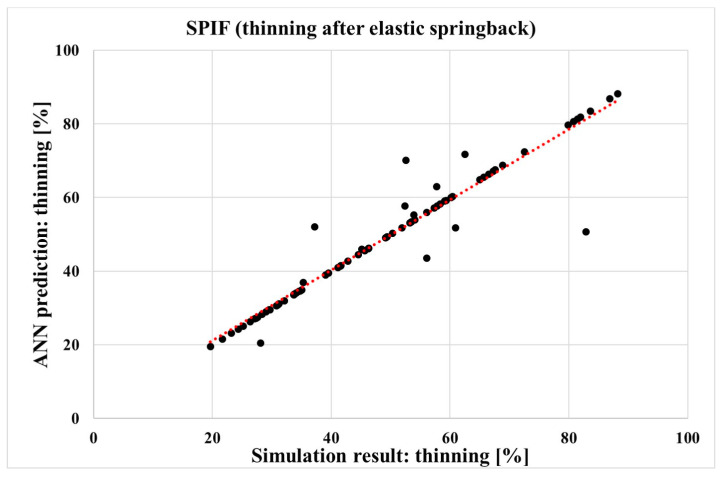
Comparison between the sheet metal thinning after elastic springback as predicted by the Multilayer Perceptron Artificial Neural Network (MLP ANN) and the thinning values obtained from simulations for single-point incremental forming (SPIF).

The MLP ANN networks were constructed based on the simulations of the forming process under consideration, with the input and corresponding output parameters listed in Table 2 and Table 4. The sheet metal thinning results from the simulations of the SPIF or hybrid two-step forming process were compared with the predictions of the MLP ANN models for the corresponding forming process and the output parameters of the sheet thinning. Figure 11 and Figure 12 show the comparison between the sheet metal thinning values predicted by the MLP ANN and the sheet metal thinning values from the simulation, either for hybrid two-step forming or for SPIF. The two diagrams show 75 points corresponding to the 75 simulations for each forming process, for the input parameter values in Table 2 and the corresponding output parameter values in Table 4.

The accuracy of the predictive ability of both MLP ANN models was first determined by calculating the correlation coefficient *R*, which was performed automatically in MATLAB for all available data together. The values of the *R* coefficient for both MLP ANN models are shown in Table 5. The values of the correlation coefficient *R* are 0.972 and 0.969. The high values of the *R* coefficient show good predictive and generalisation capabilities of the neural networks used [70]. The satisfactory predictive capabilities for both MLP ANN models are also evidenced by the regression coefficients *R*^2^ of 0.993 and 0.990, respectively, as shown in Table 5.

Correctly trained MAP ANNs thus enable the prediction of the desired output parameter value for any input parameter value. For our two trained neural networks, with all hyperparameters listed in Table 5, the predictions of sheet metal thinning for SPIF and for hybrid two-step forming can be made when all input parameter values are given to their respectable MLP ANNs. By using the same input parameter values for both neural networks, a comparison can be made between the two forming processes under consideration. With this approach, a decision can be made as to which forming process results in less sheet metal thinning for certain values of all input parameters. The prediction of output parameters using MLP ANNs and random input parameter values will be the subject of the next chapter.

When we form a desired shape of a part, we often use tools and other equipment with the geometry available to us. Most likely, the available tools and equipment are not the optimal solution for sheet metal thinning, when forming any conical part shape from a material with random material properties. For this reason, additional optimisation must be performed. Correctly set MLP ANNs can be used as a fitness function for the GA. In the following chapter, the results of using a GA to optimise the output parameters of sheet metal thinning for SPIF or hybrid two-step forming are presented. In this approach, the optimised value of the output parameter is provided, along with the input parameter values with which the optimised value was achieved. The optimised output parameter values and the corresponding input parameter values are compared with non-optimised or randomly selected input parameter values and their corresponding output parameter values. Finally, the prediction results for SPIF and hybrid two-step forming are compared, using the same input parameter values.

### 3.3. MLP ANN Prediction Results and Optimisation Results Using GA

One of the objectives of our study was to allow the user to choose the shape of a conical part and also choose the main material properties from which the final part should be made. The two MLP ANN models of sheet metal thinning for SPIF and hybrid two-step forming are based on a sufficient number of FEM simulations and allow the prediction of sheet metal thinning using arbitrary values for all tested parameters. The correctness of the MLP ANN set was discussed in the previous Section 3.2, where the value of *R*^2^ was also given. These MLP ANN models can then be used as fitness functions for optimisation with GA, with the main focus on minimising the sheet thinning as the main output parameter of the two forming processes under consideration. By using the trained MLP ANNs as the fitness function for GA, it can be assumed that the results generated by GA have the same accuracy as the respectable MLP ANN with its *R*^2^ value. During optimisation, the input parameters that define the shape of the workpiece and the material properties must be selected by the user, while all other input parameters of the study, presented in Table 1, must be optimised, with the aim of achieving the lowest possible sheet metal thinning.

For this study, randomised values for all input parameters were selected from Table 1. For this randomised selection of input parameters, the restrictions described in Section 2.1 were followed. These randomised input parameter values were then fed into the MLP ANN and the prediction of the considered output parameter was made. For this study, we decided to evaluate the developed concept with 10 randomly selected input parameter combinations or sets. Table 6 shows 10 random input parameter sets and their corresponding output parameters, for the prediction of sheet metal thinning using the MLP ANNs. Using the same input parameter values, a prediction of sheet metal thinning has been made with the MLP ANN set for hybrid two-step forming and the MLP ANN set for SPIF. The only difference is that the MLP ANN set for SPIF did not use the parameter values for the two parameters of bulging, namely the diameter of the bulging tool *D*_bulging_ and the bulging depth *H*_bulging_. The predictions for sheet metal thinning made with the set MLP ANNs provide first-hand results on the difference between hybrid two-step forming and SPIF. The preliminary work by Sevšek et al. [55] showed that the parameters’ target wall angle *α*, target workpiece height *H* and, in the case of hybrid two-step forming, the bulging depth *H*_bulging_, also have a major influence on sheet thinning, which is also evident in the results presented in Table 6. Table 6 also shows that both SPIF and hybrid two-step forming can result in lower sheet thinning with the same input parameter values. This draws attention to the complexity of making the right choice between SPIF or hybrid two-step forming, which is discussed in the rest of this chapter.

In order to achieve minimum sheet metal thinning when forming any conical product shape from a material with defined material properties, the user must first select certain input parameter values. The parameters that define the desired shape of the part and the material properties are highlighted in bold in Table 6. The values of all other input parameters must be optimised to achieve the smallest possible value of sheet metal thinning as the main output parameter of this study. GA was used for this optimisation and the results of the optimised output parameter values of sheet metal thinning, and the corresponding optimised values of the input parameters, are shown in Table 7 (for hybrid two-step forming) and in Table 8 (for SPIF). The bold input parameters and their values are the user-defined values of the parameters that define the shape of the considered conical part and the material properties. These input parameters match the input parameters marked in bold in Table 6, which contains non-optimised solutions. In both Table 7 and Table 8, the output parameter of sheet thinning was minimised for all 10 input parameter combinations, which shows that the optimisation is successful. To understand the extent of optimisation, the optimisation change is given in the last column of both Table 7 and Table 8.

A complex optimisation of sheet metal thinning during SPIF or hybrid two-step forming, considering a large number of input parameters, has led to a significant change in thinning and, thus, a more uniform sheet thickness of the final product. This optimisation applies to both forming processes under consideration, i.e., SPIF and hybrid two-step forming. As can be seen from the optimised data in Table 7 and Table 8, compared to the random initial values in Table 6, some input parameters play a more important role when it comes to minimising sheet metal thinning. In the case of hybrid two-step forming with optimised parameter values in Table 7, it is obvious that the bulging tool diameter *D*_bulging_ (in combination with the bulging depth *H*_bulging_) plays a crucial role. Although the SPIF is only performed as the second step of hybrid two-step forming, the parameters of the SPIF tool diameter D and the vertical forming step z are of great importance in the optimisation under consideration. In SPIF forming, with the optimised parameter values listed in Table 8, both the SPIF tool diameter D and the vertical forming step z play an important role in the optimisation presented. In addition, the two-dimensional parameters of the backing plate considered (*D*_tool_ and, especially, *R*_tool_) have a major influence on the thinning. This corresponds well with the preliminary study by Sevšek et al. [55], in which the influence and correlation analysis of important SPIF parameters showed the importance of the backing plate geometry when considering sheet metal thinning as the output parameter.

Due to the complexity of the presented problem, with a large number of input parameters, there is no obvious conical part shape or selected material parameters for which SPIF would be a better choice than hybrid two-step forming, when considering sheet metal thinning or vice versa. For this reason, whenever a new part shape and new material properties are selected by the user, an optimisation with a GA should be performed for both forming processes and a comparison of the sheet metal thinning and the uniformity of the sheet thickness distribution should be made.

## 4. Discussion

In our study, a multilayer perceptron artificial neural network (MLP ANN) was used for the output parameters of sheet metal thinning during single-point incremental forming (SPIF) and hybrid two-step forming, separately. The data on which the MLP ANNs were trained are among the input parameters used in the simulation models listed in Table 2 and the output parameters generated by specific simulations listed in Table 4. Correctly selected hyperparameters determine the architecture of the set MLP ANN. The hyperparameters for both MLP ANNs in this study are listed in Table 5. With these hyperparameters, their training was performed using the training data belonging to the input and output parameters of the simulations. The predictions of the neural network for sheet metal thinning were compared with the specific output parameter values of the simulations performed, examples of which are given in Figure 11 and Figure 12, for both forming processes studied. The values of the deterministic coefficient *R*^2^ are 0.972 and 0.969, respectively; this shows that the output parameter predictions and the original output parameter values produced by simulations are in good agreement.

The training of the neural networks and the predictions made using the trained neural networks were carried out on a computer with an Intel Core i7-1065G7 processor, with a clock frequency of 3.6 GHz and 16 GB RAM. Training the neural network is a lengthy process, compared to the prediction process made with a trained neural network. Training the MLP ANN for hybrid two-step forming took 21.81 s, compared to the 445.18 s needed to train the MLP ANN for SPIF. The time difference is due to the different complexity of the two trained neural networks, i.e., the different number of neurons in each of the five hidden layers. Training the MLP ANN of the two forming processes under consideration, with respect to the selected hyperparameters, is a one-time process. After the training of the specific neural network is completed, it can be used to predict the considered output parameter value using any input parameter values. The prediction process is relatively short compared to the training process. The calculations of all 10 possible input parameter combinations from Table 6 were performed separately. The times for each calculation, either with the MLP ANN for hybrid two-step forming or SPIF, are shown in Table 9. As can be seen, the average time for these 10 output parameter predictions is 0.030 and 0.028 s for hybrid two-step forming and SPIF, respectively.

For the predictions, the neural networks belonging to their respective forming process and the output parameters of the sheet metal thinning can be used. As an example, the values of the input parameters from the first row of Table 10 were entered into the MLP ANN of the hybrid two-step forming process and into the MLP ANN of SPIF, to predict the sheet metal thinning. In Table 10, the bulging tool diameter *D*_bulging_ and bulging depth *H*_bulging_ parameters are only present for hybrid two-step forming. By using MLP ANNs for both forming processes under investigation and supplying these neural networks with the same input parameter values, a comparison can be made between the two forming processes under consideration. This allows the correct forming process to be selected, which results in less sheet metal thinning and, therefore, a more uniform sheet thickness in the final part. For the example presented in the first row of Table 10, there is less sheet metal thinning with SPIF. The sheet metal thinning results for the first row of Table 10 were verified in the Abaqus simulation environment, with a sheet thinning of 41.20% being achieved for hybrid two-step forming and 39.76% for SPIF. Thus, the simulation results show minimal deviation from the MLP ANN model predictions for sheet metal thinning. The sheet metal thinning results for the verification simulations using the same input parameter values fed into the respectable MLP ANNs are shown in the last two columns of Table 10.

It should be noted that the MLP ANNs were trained using the data comprising the input parameters (Table 2) and the corresponding output parameters (Table 4), obtained from a sufficient number of simulations. The input parameters were selected within the ranges from Table 1 and the neural networks were trained on these ranges to make predictions for the given output parameters. This means that the prediction of the output parameter for which the specific MLP ANN was trained can also be made with input parameters that do not have to lie within the specified ranges. ANNs allow extrapolation, i.e., they enable predictions to be made with input parameter values that lie slightly outside the ranges for which a specific ANN was trained. Another useful property of ANNs is that they are able to generalise from the data on which they were trained. Recognising patterns created from input parameter values that are mapped to output parameter values is a feature that enables the use of wider ranges of input parameter values. The generalisation ability of neural networks can be demonstrated by a good agreement between the initial results and the predictions made by the neural network itself [36]. More complex neural networks, in terms of the number of hidden layers and corresponding neurons, can provide better generalisation capabilities. To test these two capabilities of our two neural networks, out-of-range values of the input parameters were used and the values of the output parameters of sheet metal thinning were predicted. The out-of-range input parameter values and the corresponding output parameter values are listed in Table 10, starting from the second row. The input parameter values have been changed compared to the first row of input parameter values in Table 10. Input parameter values that lie outside the specified ranges of the input parameter values from Table 1, on which the neural networks in this study were trained, are marked in bold. The results of the MLP ANN prediction are compared with the results of the simulations using the same input parameter values written for both analysed forming processes in the last two columns of Table 10. Some changes in the input parameters have a greater influence on sheet metal thinning than others. According to the study by Sevšek et al. [55] that focused on the influence analysis using the random forest method, we can emphasise the parameters of the desired wall angle *α*, the target workpiece height *H,* and also the inlet radius of the backing plate *R*_tool_. In addition, the bulging tool diameter *D*_bulging_, combined with the bulging depth *H*_bulging_ and SPIF tool diameter *D*, play an important role [55]. The pre-bulging phase with the correct choice of bulging depth and bulging tool diameter can thus minimise sheet metal thinning compared to SPIF as a stand-alone process. It should be emphasised that choosing an incorrect value for the bulging depth or bulging tool diameter can also worsen the sheet metal thinning. For this reason, an optimisation approach using a genetic algorithm (GA) is implemented in our study.

**Figure 13:**
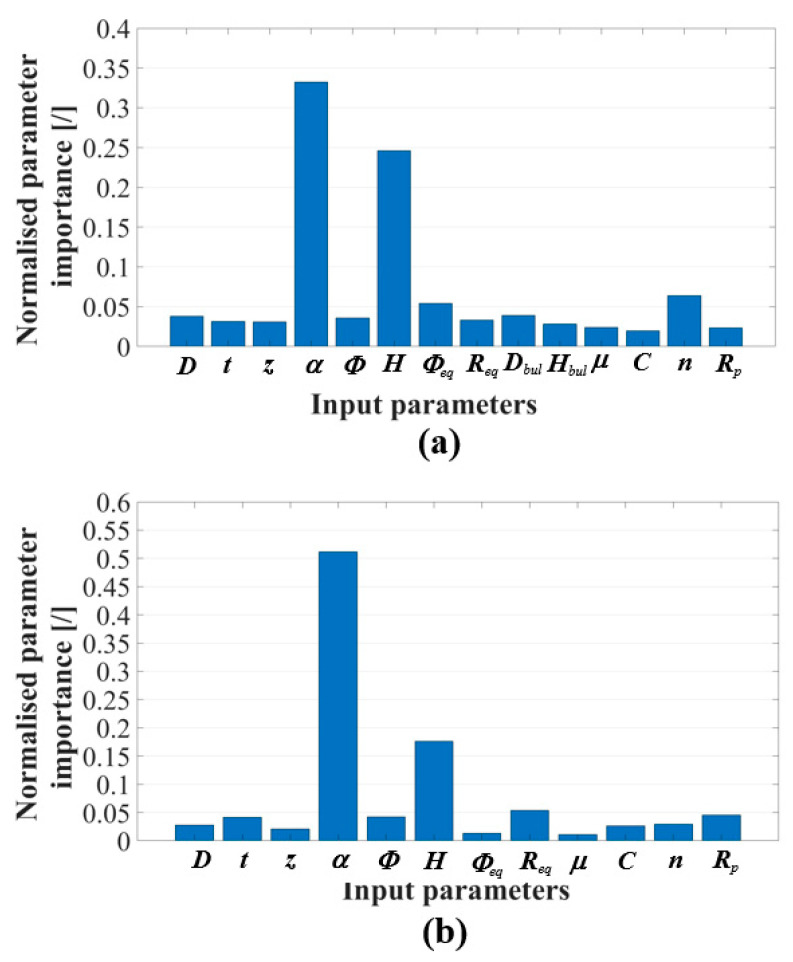
Individual influence of the input parameters on the output parameter of sheet metal thinning for (**a**) hybrid two-step forming and (**b**) single-point incremental forming (SPIF).

To illustrate this difference in influence between 12 different input parameters of the SPIF and 14 different input parameters of the hybrid two-step forming, the set neural networks of both forming processes were used as the basis for the influence analysis. The permutation feature importance approach was implemented in MATLAB, tailoring the code to handle 12 and 14 input parameters. In this approach, each input parameter is varied independently and the resulting increase in prediction error is the basis for calculating the importance of that specific input parameter. The influence results shown in Figure 13 are normalised to sum to 1, making the results easy to interpret. A change in the values of input parameters, whose influence on the output parameter is large, leads to a drastic change in the value of the analysed output parameter. For both SPIF and hybrid forming, we can emphasise the target wall angle and the target part height as significant influencing parameters. Other input parameters have less influence on sheet metal thinning, but their influence should still not be overlooked. These include the backing plate diameter and lead-in radius, the diameter of the bulging tool, and the depth of bulging in hybrid forming, as well as the lead-in radius of the backing plate, the initial diameter of the conical test piece, and the initial sheet thickness in SPIF. In contrast, some material parameters and the coefficient of friction only have a minor influence in hybrid forming. The coefficient of friction and the inner diameter of the backing plate also have a minor influence on the thinning during SPIF. If the values of these more influential input parameters lie outside the range in which the neural network was trained, then the predictions of the set MLP ANN can deviate considerably from the results of the simulations with the same input parameter values. The same applies if all input parameter values lie outside the range for which the neural network was trained. This can be seen from the prediction results and the corresponding simulation results in Table 10.

**Figure 14:**
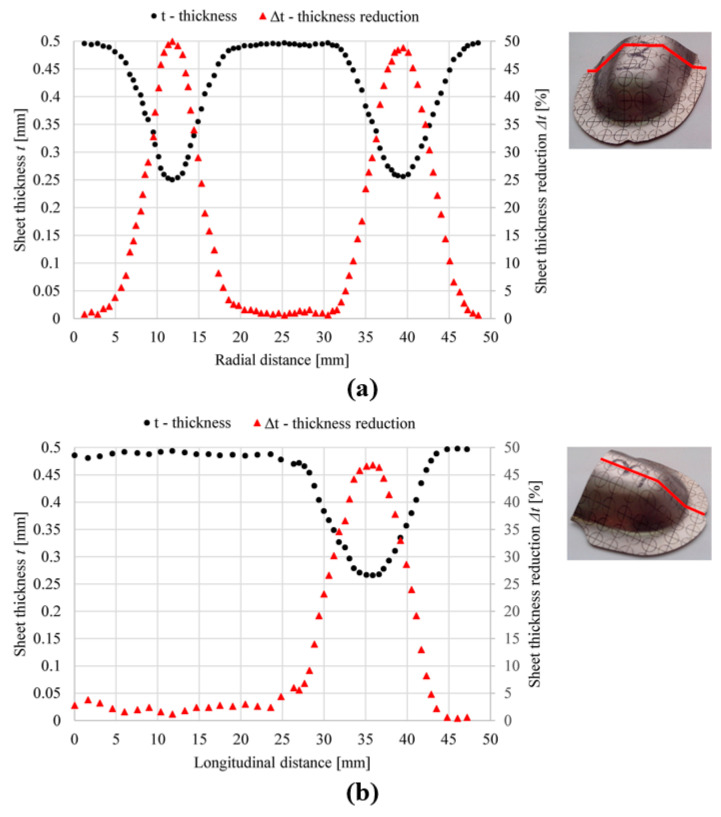
Denture from the study by Milutinović et al. [71] formed with single-point incremental forming (SPIF). The sheet thickness was measured in (**a**) transverse direction and (**b**) longitudinal direction.

The MLP ANN models for both SPIF and hybrid two-step forming were built on data provided by simulations of forming towards a conical shape of the final product. This type of geometry is commonly used in SPIF studies as it is the standard way to test the influence of parameters. Nevertheless, the models of this study can be used to predict sheet metal thinning during the forming of a more complex geometry. This additional validation was performed using a geometry from the study by Milutinović et al. [71], which had a more complicated shape compared to the conical geometry. In the said study, a stainless-steel sheet of X6Cr17 with a thickness of 0.5 mm was formed using SPIF to the final shape of a denture which is shown in Figure 14. The tensile test provided the essential material data for the strength coefficient *C* of 932.9 MPa, the work hardening coefficient *n* of 0.219, and the yield strength *R*_p_ of 318.7 MPa. The final depth of 14 mm was achieved with a vertical forming step of 0.02 mm. The backing plate without entry radius with an axiasymetric opening was used, having a radius of 20 mm at the front wall or at the shorter side of the denture and 74 mm at the side wall or at the longer side of the denture. Forming was performed with a hemispherical tool with a diameter of 10 mm. The forming process was aimed towards a front wall angle of 45° and a side wall angle of 53°. Despite this being an axiasymetric final product with a variation of the wall angle, the prediction with the MLP ANN set for SPIF presented in our study still provides satisfactory results. The prediction result of sheet metal thickness reduction is 49.2% for the front wall or transverse direction (Figure 14a) and 45.08% for the side wall or longitudinal direction (Figure 14b). The prediction results presented in our study using MLP ANN show good agreement with the experimental results presented in the study by Milutinović et al. [71].

In addition, the predictive abilities of the two MLP ANNs were tested using data from the studies by Mulay et al. [23] and Bansal et al. [69]. Both studies focussed on SPIF forming, with some parameter values falling outside the ranges shown in Table 1. In the study by Mulay et al. [26], SPIF was performed with a hemispherical tool of 10 mm diameter on DC04 steel and AA5754-H22 aluminium, both with an initial sheet thickness of 0.8 mm. Apart from the material parameters not belonging to the DP-type steel and the initial sheet thickness being outside the MLP ANN training range, the main problem in predicting sheet metal thinning with SPIF’s MLP ANN model was the geometry of the conical final product. The height and initial diameter of the conical product in Mulay et al. [23] was 80 mm and 118 mm, respectively. Both values are outside the parameter ranges in Table 1, on which the MLP ANN belonging to SPIF was trained. Nevertheless, the predictions of the MLP ANN model were within 5% of the actual sheet thickness change reported in the study by Mulay et al. [23]. It should be noted that the initial part diameter plays a less important role when it comes to sheet metal thinning [55]. Additionally, despite a large difference between the target value of the product height of 80 mm and the maximum value of this parameter of 40 mm, on which the MLP ANN was still trained, there is a uniform sheet thickness change in the wall area. This uniformity of the sheet thickness on the wall area coincides well with the sinusoidal prediction of the final sheet thickness presented in the study by Mulay et al. [23]. Similar observations can be made for the predictions using the MLP ANN belonging to SPIF, which were performed using parameter values from the study by Bansal et al. [69]. SPIF was performed on a 0.88 mm thick AA5052-O aluminium sheet, to obtain the final conical shapes of parts with different wall angles, formed with different diameters of a hemispherical tool and using different values for the vertical forming step [69]. For the target wall angle of 45°, the MLP ANN predictions were found to be in good agreement with the actual values of final sheet thickness reported by Bansal et al. [69]; the discrepancy between predicted and actual values was less than 6%. The main reason for this discrepancy could be that the initial sheet thickness and material property values used to train the MLP ANN were outside the training range. A larger deviation between the sheet thinning predicted by MLP ANN and the actual sheet thinning reported by Bansal et al. [69] was present at a target wall angle of 22°. The deviation of almost 17% is due to the fact that the target wall angle is outside the range for which the MLP ANN was trained (see Table 1) and that the target wall angle parameter has a greater influence on the sheet thickness change, as reported by Sevšek et al. [55].

A GA was used to minimise the output parameter of sheet metal thinning and to determine the corresponding input parameter values. The main objective of this study was to allow the user to select the values of the parameters that determine the shape of the desired part, as well as the parameters that determine the material properties and the initial thickness of the sheet metal used, while changing all other input parameters to optimise the sheet metal thinning. The parameters chosen by the users were the initial sheet metal thickness *t*_0_, the target wall angle of the conical part *α*, the initial tool path diameter *D*_path_, the target part height *H*, the yield strength *R*_p_, the strength coefficient *C,* and the strain hardening coefficient *n*. All the input parameters mentioned, and the parameters that are optimised in the study, are presented in Table 1. The optimisation process using a GA provides the optimised values of the other input parameters, with which minimum sheet metal thinning is possible (with the exception of those selected by the user). The fitness functions used for the optimisation process were the MLP ANNs associated with the two forming processes being studied and the sheet metal thinning output parameter. The optimisation process, using the GA of the study presented here, works with a population size of 10 and a number of generations of 100. The duration of the optimisation process depends on the iteration and usually takes a few minutes.

**Figure 15:**
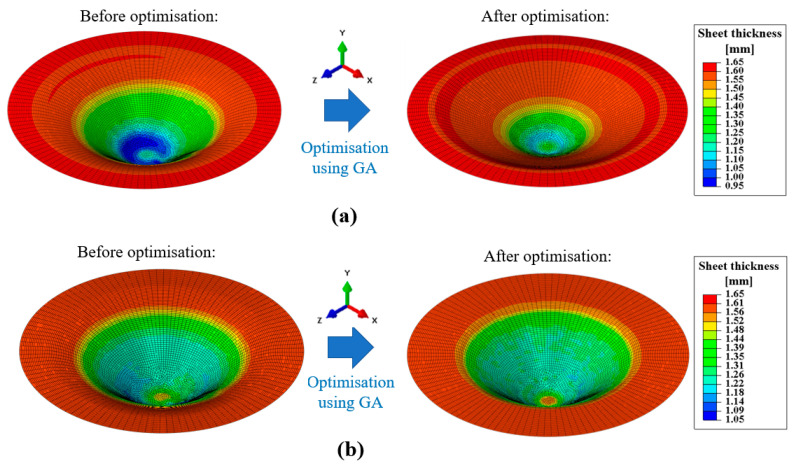
Simulation results using the randomised values of input parameters belonging to the fifth row of Table 6 for (**a**) hybrid two-step forming and (**b**) single-point incremental forming (SPIF). Comparison with the simulation results using the optimised values of input parameters belonging to the fifth row of Table 7 for (**a**) hybrid two-step forming and the fifth row of Table 8 for (**b**) SPIF, both with an initial sheet thickness of 1.6 mm.

To further demonstrate the capabilities of the presented optimisation algorithm, additional simulations were performed with the randomised input parameters from the fifth row of Table 6, for both SPIF and hybrid two-step forming. In addition, simulations were carried out with the optimised input parameter values for hybrid two-step forming and for SPIF. The input parameters from the fifth row of Table 7 and Table 8 were considered. Using the fifth row of random parameters from Table 6, the repeated FEM simulations of the hybrid two-step process resulted in the final sheet metal thickness value of 0.951 mm, which corresponds to a thinning of 40.56%. Using the random input parameter values from the first row of Table 6, the repeated FEM simulations of the SPIF process resulted in a final sheet metal thickness value of 1.084 mm, corresponding to a thinning of 32.25%. Using the optimised values of the input parameters from the fifth row of Table 7, for hybrid two-step forming, and the fifth row of Table 8, for SPIF, the FEM simulations resulted in final sheet thicknesses of 1.122 mm and 1.150 mm, respectively. These two values result in a thinning of 29.88% and 28.13%, respectively. The results of these repeated simulations agree well with the results presented in Table 6, Table 7 and Table 8, with a deviation of less than 2% in the predicted sheet metal thinning for all cases presented. The main simulation results discussed in this section are shown in Figure 15. It should be emphasised that all input parameters defining the shape of the conical part (*α*, *D*_path_, *H*), the material parameters (*C*, *n*, *R*_p_), and the initial sheet thickness (*t*_0_) are user-defined and, therefore, the same, for all four cases presented. It is obvious that the optimisation of only some input parameters (*D*, *z*, *D*_tool_, *R*_tool_, *D*_bulging_, *H*_bulging_, *μ*) can minimise the sheet metal thinning and generate a more uniform distribution of the final thickness of the workpiece. It is worth noting that, in a real environment, some input parameters can be changed more easily than others, while the input parameters that define the part geometry, tool geometry, equipment geometry, and sheet properties remain unchanged. Two such examples are the vertical forming step parameter *z* and the bulging depth *H*_bulging_.

## 5. Conclusions

In this study, we developed and validated predictive models using multilayer perceptron artificial neural networks (MLP ANNs) for single-point incremental forming (SPIF) and a hybrid two-step forming process combining bulging and SPIF. These models were trained on data from finite element method (FEM) simulations to predict sheet metal thinning accurately. Key findings and contributions of the study are as follows:

1. Predictive capability: The MLP ANN models demonstrated high predictive accuracy for sheet metal thinning in both SPIF and hybrid two-step forming processes. This capability allows for reliable predictions based on various geometric, technological, and material parameters. A comparison between the original simulation results and the results predicted by the corresponding neural network shows a small deviation affecting the *R*^2^ values, which are close to 1. The *R*^2^ values of the neural networks belonging to the output parameter of sheet metal thinning of the SPIF and hybrid two-step forming process are 0.993 and 0.990, respectively.
2. Optimisation using genetic algorithm (GA)**:** By employing a GA with the trained MLP ANNs as fitness functions, we optimised the forming process parameters to minimise sheet metal thinning. This approach provided an average improvement of 21.95% for hybrid two-step forming and 16.23% for SPIF.
3. Comprehensive parameter consideration: This study uniquely considers a wide range of parameters (technological, geometrical, and material) simultaneously, which has not been previously addressed. This comprehensive approach enhances the robustness of the predictive models.
4. User-defined flexibility: The models allow users to select specific geometric and material parameters while optimising other parameters to achieve minimal thinning. This flexibility makes the models practical for various industrial applications and opens up new possibilities for further research in the field of sheet metal forming.
5. Comparison of forming processes: The developed models enable a comparative analysis between SPIF and hybrid two-step forming, allowing users to select the most suitable process for achieving lower sheet metal thinning and the desired conical shape of the product from the sheet metal with defined material properties and initial sheet thickness.

Beyond the specific results, this study contributes to a fundamental understanding of how different parameters interact to influence sheet metal thinning in flexible forming processes. The methods developed here can be generalised to other materials and forming scenarios, making them relevant to a wide range of applications beyond the current study. This work advances the academic field by providing a framework for the optimisation of forming processes that can be adapted to different materials, including advanced high-strength steels and other challenging alloys. The knowledge gained from this research can also be used in the development of more efficient and precise manufacturing processes, paving the way for further advances in Industry 4.0 environments. Future work will focus on evaluating additional input parameters, such as material anisotropy and different tool path strategies of SPIF, to further improve the accuracy and applicability of the models. Furthermore, we will investigate the geometric accuracy of the formed parts and integrate in-process adjustments for real-time optimisation in an Industry 4.0 context.

In addition to the technical advances presented, this study also has a significant economic impact on the industry, which relies on flexible forming processes. The ability to predict and minimise sheet thinning through MLP ANN and GA-driven optimisation directly reduces material waste, which is especially valuable when forming high-cost materials such as advanced high-strength steels (AHSSs). With more accurate thickness distributions, manufacturers can reduce reliance on post-processing steps, lowering production costs and energy consumption associated with reworking or scrapping non-conforming parts. In addition, the predictive capabilities developed in this study can help reduce tooling adjustments and downtime, ultimately increasing production throughput. For small batch or custom production, especially in the automotive and aerospace industries, this method is a cost-effective solution as it enables rapid parameter adjustments without extensive physical trials. Reducing trial-and-error iterations not only minimises labour and material costs, but also shortens time-to-market, increasing the competitiveness and sustainability of production systems using this approach.

## Figures and Tables

**Table 1:** Parameter symbol, name, and units used, with the maximum and minimum values used in this study.

Parameter Symbol	Parameter Name	Parameter Unit	Minimum	Maximum
*D*	SPIF tool diameter	[mm]	5	15
*t*_0_	Initial sheet thickness	[mm]	1	4
*z*	Vertical forming step	[mm]	0.1	1
*α*	Target conical workpiece wall angle	[°]	30	80
*D*_path_	Initial tool path diameter	[mm]	50	80
*H*	Target conical workpiece height	[mm]	10	40
*D*_tool_	Inner backing plate diameter	[mm]	50	90
*R*_tool_	Backing plate inlet radius	[mm]	2.5	10
*D*_bulging_	Bulging tool diameter	[mm]	20	50
*H*_bulging_	Bulging depth	[mm]	10	40
*μ*	Coefficient of friction	[/]	0.05	0.2
*C*	Strength coefficient	[MPa]	900	1400
*n*	Strain-hardening coefficient	[/]	0.14	0.24
*R*_p_	Yield strength	[MPa]	330	760

**Table 2:** Parameter values in 75 simulations of single-point incremental forming (SPIF) and 75 simulations of hybrid two-step forming that uses additional parameters of bulging tool diameter and bulging depth.

	Simulation Parameters													
Simulation Number	*D* [mm]	*t* [mm]	*z* [mm]	*α* [°]	*D*_path_ [mm]	*H* [mm]	*D*_tool_ [mm]	*R*_tool_ [mm]	*D*_bulging_ [mm]	*H*_bulging_ [mm]	*μ* [/]	*C* [MPa]	*n* [/]	*R*_p_ [MPa]
1	14.4	3.9	0.1	45	65	12	80	4.9	34	11	0.06	1081	0.22	332
2	13.8	3.9	0.3	69	51	30	82	9.5	28.9	28	0.07	902	0.15	367
3	8.9	3	0.2	38	62	14	82	2.9	30.6	10	0.18	1030	0.24	417
…	…	…	…	…	…	…	…	…	…	…	…	…	…	…
75	11	1.6	1	60	70	27	77	3.6	23.4	22	0.08	1127	0.23	365

**Table 3:** Number of quadrilateral and triangular elements on individual 3D objects of the simulation models used in this study.

3D Object	Number of Quadrilateral S4R or R3D4 Elements	Number of Triangular S3 or R3D3 Elements
Sheet metal blank	8233	52
Backing plate	1400–2250	20–50
Blank holder	2000–4050	50–100
SPIF tool	1000–1600	5–10
Bulging tool	5000–8000	150–300

**Table 4:** Simulation results of sheet metal thinning after elastic springback for hybrid two-step forming and single-point incremental forming (SPIF).

	Hybrid Two-Step Forming	SPIF
Simulation Number	Δ*t* [%]	Δ*t* [%]
1	23.54	25.17
2	59.87	82.87
3	28.28	24.35
…	…	…
75	58.69	60.39

**Table 5:** Characteristics of multilayer perceptron artificial neural network (MLP ANN) architectures for the output parameters of sheet metal thinning after elastic springback (Δ*t*) for hybrid two-step forming and single-point incremental forming (SPIF).

Output	*R*	*R* ^2^	Hidden Layer Sizes	Activation Function	Solver	Initial Learning Rate	Learning Rate Type	L2
Δ*t* [%]—Hybrid forming	0.972	0.993	(20 20 20 20 20)	sigmoid	adam	0.0001	constant	0.0100
Δ*t* [%]—SPIF	0.969	0.990	(100 80 60 40 20)	sigmoid	adam	0.0100	adaptive	0.0001

**Table 6:** Randomised input parameter values and the predicted output parameter values of sheet metal thinning using the multilayer perceptron artificial neural network (MLP ANN) set for hybrid two-step forming and single-point incremental forming (SPIF). User defined input parameters are marked in bold.

	Input Parameters														Hybrid Two-Step Forming	SPIF
Number	*D* [mm]	*t* [mm]	*z* [mm]	*a* [°]	*D*_path_ [mm]	*H* [mm]	*D*_tool_ [mm]	*R*_tool_ [mm]	*D*_bulging_ [mm]	*H*_bulging_ [mm]	*μ* [/]	*C* [MPa]	*n* [/]	*R*_p_ [MPa]	Δ*t* [%]	Δ*t* [%]
1	13.7	**1.5**	0.1	**65**	**75**	**17**	86	7.2	20.4	11	0.13	**1221**	**0.23**	**363**	69.86	77.79
2	11.8	**3.8**	0.1	**46**	**62**	**16**	70	3.9	21.2	12	0.15	**1041**	**0.22**	**371**	45.26	41.12
3	13.8	**2.2**	0.25	**33**	**58**	**16**	86	2.7	46.8	13	0.18	**1234**	**0.18**	**469**	22.89	30.22
4	9.7	**3.9**	0.2	**52**	**61**	**17**	86	2.7	31.3	16	0.19	**1144**	**0.17**	**532**	31.08	42.53
5	14.9	**1.6**	1	**37**	**72**	**24**	79	9.1	26.8	17	0.13	**1225**	**0.18**	**544**	39.21	31.73
6	14.4	**3.4**	0.5	**74**	**65**	**20**	81	4.8	25.8	12	0.12	**945**	**0.16**	**350**	62.74	66.48
7	10.1	**1**	0.25	**58**	**57**	**11**	72	7.9	49.7	10	0.11	**1284**	**0.17**	**393**	28.66	38.05
8	12.2	**2.9**	0.25	**64**	**54**	**35**	77	4.2	37.9	30	0.17	**967**	**0.22**	**390**	61.91	83.59
9	11.2	**2.1**	0.1	**44**	**52**	**24**	81	6.9	23.1	22	0.08	**1142**	**0.24**	**407**	58.71	47.15
10	8.8	**3.7**	0.5	**57**	**59**	**23**	84	9.1	22.9	11	0.18	**972**	**0.18**	**405**	51.66	61.22

**Table 7:** Randomly selected and optimised input parameter values, optimised value of sheet metal thinning, and the optimisation change for hybrid two-step forming. User-defined input parameters are marked in bold.

Number	*D* [mm]	*t* [mm]	*z* [mm]	*α* [°]	*D*_path_ [mm]	*H* [mm]	*D*_tool_ [mm]	*R*_tool_ [mm]	*D*_bulging_ [mm]	*H*_bulging_ [mm]	*μ* [/]	*C* [MPa]	*n* [/]	*R*_p_ [MPa]	Δ*t* [%]	Optimisation Change of Δ*t* [%]
1	5.05	**1.5**	0.405	**65**	**75**	**17**	81.34	2.5	39.4	17	0.20	**1221**	**0.23**	**363**	46.53	23.33
2	10.78	**3.8**	0.364	**46**	**62**	**16**	90.00	9.3	46.0	16	0.06	**1041**	**0.22**	**371**	19.79	25.47
3	5.13	**2.2**	0.178	**33**	**58**	**16**	63.99	9.8	33.0	16	0.06	**1234**	**0.18**	**469**	14.66	8.24
4	6.16	**3.9**	0.327	**52**	**61**	**17**	76.45	9.6	50.0	17	0.06	**1144**	**0.17**	**532**	17.09	13.99
5	8.97	**1.6**	0.421	**37**	**72**	**24**	89.83	5.3	46.4	24	0.19	**1225**	**0.18**	**544**	28.65	10.56
6	15.00	**3.4**	0.385	**74**	**65**	**20**	90.00	10.0	50.0	20	0.06	**945**	**0.16**	**350**	33.78	28.97
7	8.55	**1**	0.157	**58**	**57**	**11**	68.07	10.0	42.2	11	0.06	**1284**	**0.17**	**393**	15.40	13.25
8	14.98	**2.9**	0.365	**64**	**54**	**35**	90.00	9.0	50.0	35	0.18	**967**	**0.22**	**390**	30.81	31.10
9	14.99	**2.1**	0.364	**44**	**52**	**24**	86.42	10.0	50.0	24	0.19	**1142**	**0.24**	**407**	24.99	33.72
10	14.64	**3.7**	0.247	**57**	**59**	**23**	90.00	9.4	50.0	23	0.06	**972**	**0.18**	**405**	20.75	30.91
															Average:	21.95

**Table 8:** Randomly selected and optimised input parameter values, optimised value of sheet metal thinning, and the optimisation change for single-point incremental forming (SPIF). User-defined input parameters are marked in bold.

Number	*D* [mm]	*t* [mm]	*z* [mm]	*α* [°]	*D*_path_ [mm]	*H* [mm]	*D*_tool_ [mm]	*R*_tool_ [mm]	*D*_bulging_ [mm]	*H*_bulging_ [mm]	*μ* [/]	*C* [MPa]	*n* [/]	*R*_p_ [MPa]	Δ*t* [%]	Optimisation Change of Δ*t* [%]
1	5.00	**1.5**	0.333	**65**	**75**	**17**	75.00	2.7	/	/	0.20	**1221**	**0.23**	**363**	50.73	27.06
2	10.52	**3.8**	0.340	**46**	**62**	**16**	80.60	4.2	/	/	0.05	**1041**	**0.22**	**371**	25.88	15.25
3	10.79	**2.2**	0.222	**33**	**58**	**16**	85.20	8.9	/	/	0.19	**1234**	**0.18**	**469**	8.80	21.42
4	5.26	**3.9**	0.181	**52**	**61**	**17**	62.60	9.8	/	/	0.11	**1144**	**0.17**	**532**	26.85	15.68
5	6.60	**1.6**	0.247	**37**	**72**	**24**	75.86	2.5	/	/	0.20	**1225**	**0.18**	**544**	28.72	3.02
6	5.00	**3.5**	0.278	**74**	**65**	**20**	87.64	3.4	/	/	0.05	**945**	**0.16**	**350**	46.00	20.48
7	6.76	**1**	0.164	**58**	**57**	**11**	81.85	4.2	/	/	0.20	**1284**	**0.17**	**393**	26.34	11.72
8	15.00	**2.9**	0.745	**64**	**54**	**35**	54.01	9.0	/	/	0.05	**967**	**0.22**	**390**	58.84	24.75
9	5.00	**2.1**	0.304	**44**	**52**	**24**	61.26	2.5	/	/	0.05	**1142**	**0.24**	**407**	39.94	7.21
10	9.67	**3.7**	0.469	**57**	**59**	**23**	87.33	4.0	/	/	0.05	**972**	**0.18**	**405**	45.54	15.68
															Average:	16.23

**Table 9:** Computation time of predictions using multilayer perceptron artificial neural network (MLP ANN) of hybrid two-step forming and MLP ANN of single-point incremental forming (SPIF) using input parameter values from Table 6.

Iteration of Computation	Computation Time for One Prediction—MLP ANN of Hybrid Two-Step Forming [s]	Computation Time for One Prediction—MLP ANN of SPIF [s]
1	0.027	0.024
2	0.027	0.028
3	0.025	0.022
4	0.028	0.027
5	0.029	0.027
6	0.029	0.031
7	0.033	0.031
8	0.033	0.030
9	0.038	0.032
10	0.033	0.030
Average time for one prediction [s]:	0.030	0.028

**Table 10:** Randomly selected input parameter values and the predictions of sheet metal thinning made with the multilayer perceptron artificial neural network (MLP ANN) of hybrid two-step forming or MLP ANN of single-point incremental forming (SPIF). Comparison of the prediction results of MLP ANNs with the results of the simulations of the hybrid two-step forming and SPIF with same input parameter values. The values marked in bold are outside the ranges for which the MLP ANNs were trained.

	Input Parameters														Hybrid Two-Step Forming—MLP ANN	SPIF—MLP ANN	Hybrid Two-Step Forming—Simulation	SPIF—Simulation
Number	*D* [mm]	*t* [mm]	*z* [mm]	*a* [°]	*D*_path_ [mm]	*H* [mm]	*D*_tool_ [mm]	*R*_tool_ [mm]	*D*_bulging_ [mm]	*H*_bulging_ [mm]	*μ* [/]	*C* [MPa]	*n* [/]	*R*_p_ [MPa]	Δ*t* [%]	Δ*t* [%]	Δ*t* [%]	Δ*t* [%]
1 *	10	1	0.1	45	70	25	90	5	50	15	0.1	900	0.14	390	43.46	41.43	41.20	39.76
2	10	1	0.25	45	70	20	90	5	50	20	0.1	**874**	**0.12**	391	38.28	38.49	35.28	36.58
3	10	1	0.25	45	70	20	90	5	20	20	0.1	**874**	**0.12**	391	52.34	38.49	51.32	36.58
4	10	1	0.25	45	70	20	90	5	50	**5**	0.1	**874**	**0.12**	391	37.27	38.49	35.01	36.58
5	10	1	0.25	45	70	20	**95**	5	50	20	0.1	**874**	**0.12**	391	37.85	38.40	33.64	35.25
6	**20**	**4.2**	0.25	45	70	20	90	5	50	20	0.1	**874**	**0.12**	391	19.90	26.20	21.52	32.21
7	**4**	**0.9**	0.2	46	**84**	21	**95**	5	**52**	20	0.1	**874**	**0.12**	391	33.96	37.31	31.00	34.00
8	**4**	**0.9**	**0.09**	**28**	**84**	**9**	**95**	**2**	**56**	**7**	**0.22**	**874**	**0.12**	**321**	27.61	26.29	18.56	11.89

* All input parameter values are within the ranges on which the MLP ANNs were trained.

## Data Availability

The original contributions are presented in the article; further inquiries can be addressed to the corresponding author.
